# Bi-FoRe: an efficient bidirectional knockin strategy to generate pairwise conditional alleles with fluorescent indicators

**DOI:** 10.1007/s13238-020-00747-1

**Published:** 2020-07-17

**Authors:** Bingzhou Han, Yage Zhang, Xuetong Bi, Yang Zhou, Christopher J. Krueger, Xinli Hu, Zuoyan Zhu, Xiangjun Tong, Bo Zhang

**Affiliations:** 1 Key Laboratory of Cell Proliferation and Differentiation of the Ministry of Education, Peking University Genome Editing Research Center, College of Life Sciences, Peking University, Beijing, 100871 China; 2 School of Biology and Biological Engineering, South China University of Technology, Guangzhou, 510006 China; 3 Department of Biomedical Engineering, College of Engineering, Peking University, Beijing, 100871 China; 4 Wallace H. Coulter Department of Biomedical Engineering, Georgia Institute of Technology and Emory, Atlanta, GA 33032 USA; 5 Institute of Molecular Medicine, Peking University, Beijing, 100871 China

**Keywords:** CRISPR/Cas, conditional knockout, allele labeling, conditional rescue, minicircle DNA

## Abstract

Gene expression labeling and conditional manipulation of gene function are important for elaborate dissection of gene function. However, contemporary generation of pairwise dual-function knockin alleles to achieve both conditional and geno-tagging effects with a single donor has not been reported. Here we first developed a strategy based on a flipping donor named FoRe to generate conditional knockout alleles coupled with fluorescent allele-labeling through NHEJ-mediated unidirectional targeted insertion in zebrafish facilitated by the CRISPR/Cas system. We demonstrated the feasibility of this strategy at *sox10* and *isl1* loci, and successfully achieved Cre-induced conditional knockout of target gene function and simultaneous switch of the fluorescent reporter, allowing generation of genetic mosaics for lineage tracing. We then improved the donor design enabling efficient one-step bidirectional knockin to generate paired positive and negative conditional alleles, both tagged with two different fluorescent reporters. By introducing Cre recombinase, these alleles could be used to achieve both conditional knockout and conditional gene restoration in parallel; furthermore, differential fluorescent labeling of the positive and negative alleles enables simple, early and efficient real-time discrimination of individual live embryos bearing different genotypes prior to the emergence of morphologically visible phenotypes. We named our improved donor as Bi-FoRe and demonstrated its feasibility at the *sox10* locus. Furthermore, we eliminated the undesirable bacterial backbone in the donor using minicircle DNA technology. Our system could easily be expanded for other applications or to other organisms, and coupling fluorescent labeling of gene expression and conditional manipulation of gene function will provide unique opportunities to fully reveal the power of emerging single-cell sequencing technologies.

**Electronic supplementary material:**

The online version of this article (10.1007/s13238-020-00747-1) contains supplementary material, which is available to authorized users.

## Introduction

Complex genome modification techniques utilizing site-specific knockin (KI) to achieve gene expression labeling by protein tags or gene inactivation or reactivation *via* Cre/*loxP* system have advanced remarkably since the emergence of engineered endonucleases such as TALENs and the CRISPR/Cas system (Zu et al., 2013; Auer et al., 2014; Shin et al., 2014; Li et al., 2015; Hoshijima et al., 2016; Sugimoto et al., 2017; Burg et al., 2018; Luo et al., 2018; Li et al., 2019, 2020). These technical developments are particularly important in organisms lacking embryonic stem cell-based approaches, including zebrafish (*Danio rerio*). Li et al. reported a fluorescent gene-tagging method which introduced a fluorescent reporter into the last intron via nonhomologous end joining (NHEJ)-mediated targeted insertion in zebrafish using the CRISPR/Cas system (Li et al., 2015), taking advantage of its higher KI efficiency than homologous recombination (HR) (Auer et al., 2014). Recently, we and others further improved the technique and established “two-in-one” dual-function KI strategies to achieve conditional knockout (CKO) coupled with in-frame fusion of double fluorescent reporters to label two different alleles (positive/normal vs. negative/defective), facilitated by specially designs of dual-cassette donors (Li et al., 2019, 2020). However, generation of conditional rescue alleles through these new approaches has not been reported, and a method for simultaneously generating CKO and conditional rescue allele pairs is also not available. Furthermore, a method which could simultaneously generate fluorescent labeling of both positive and negative conditional alleles in the same target gene through a single experiment has not been established.

Unlike HR, integration of donor vectors into the host genome through the NHEJ pathway theoretically could happen in two directions, though KI in only one particular direction is useful for the previously reported methods. We sought to take full advantage of high-efficient targeted insertion and designed a versatile flipping donor vector called FoRe (forward and reverse), and its improved version called Bi-FoRe, to achieve pairwise and dual-function genome modifications which couple fluorescent labeling of gene expression with conditional manipulation of gene function, in both positive and negative states, via NHEJ-mediated targeted insertion. Two functional components, the positive Forward component and the negative Reverse component, were designed in each of these vectors to maintain and disrupt target gene functions, respectively. The two components were arranged in an opposite orientation, so that positive/normal and negative/defective alleles could be generated through forward and reverse integrations, respectively, within a single KI experiment. In addition, to achieve allele-labeling, two different fluorescent reporter genes, *tdTomato* and *EGFP*, were included and fused in-frame with the Forward component and the Reverse component, respectively. Furthermore, the two components were flanked by oppositely oriented *lox66* and *lox71* sequences, so that CKO and conditional rescue could be achieved through Cre recombinase-induced inversion of the two components in the positive/normal and negative/defective alleles, respectively. The feasibility of the FoRe and the Bi-FoRe strategies were evaluated at the *sox10* and *isl1* loci and demonstrated at the *sox10* locus, respectively.

As retention of the bacterial sequence from the donor vector within the KI allele may interfere with the expression or function of the two functional components, we constructed the Bi-FoRe donor vector using a minicircle plasmid whose bacterial backbone is flanked by minimal *attB*/*attP* sites and could be removed by phiC31 integrase, either *in vitro* or *in vivo*. We successfully achieved efficient *in vivo* elimination of the plasmid backbone through injection of *phiC31* mRNA into embryos bearing KI alleles derived from the insertion of the original full length Bi-FoRe donor. We also constructed a backbone-free minicircle Bi-FoRe donor by *in vitro* removal of the bacterial sequence using minicircle DNA production technology before injection of the donor vector into embryos and demonstrated its high efficiency in the generation of KI alleles.

## Results

### Rationale and design of the bidirectional dual-function FoRe donor to generate positive and negative conditional alleles coupled with allele-labeling through targeted insertion

To generate both positive and negative conditional alleles in parallel within one experiment, and simultaneous tagging with different fluorescent reporter genes, we designed a bidirectional KI donor consisting of two major functional components, positive and negative, in a back-to-back opposite orientation, to create and label positive/normal alleles and negative/defective alleles, respectively (Fig. S1). In addition, a *lox66* site and a *lox71* site were included in each of these two components, respectively, also in an opposite orientation (Albert et al., 1995), to enable Cre-dependent conditional inversion of the positive allele to achieve CKO effect and of the negative allele to achieve conditional rescue effect, respectively (Fig. S1). The two floxed components were placed downstream of a highly efficient CRISPR/Cas9 target site from the human *EMX1* (*hEMX1*) gene to facilitate *in vivo* linearization of the donor vector (Fig. S1; Table S1). Theoretically, this donor vector could be introduced into the CRISPR/Cas9 site in the target gene through targeted insertion in either orientation via the NHEJ pathway activated by the CRISPR/Cas9 system (Fig. S1) (Lin et al., 2014). Insertion leading to a positive allele is considered forward integration while insertion leading to a negative allele is considered reverse integration. Accordingly, the two components were designated as the Forward (Fo) component and the Reverse (Re) component, and the donor was thus named FoRe, and the region covering the two components was called the FoRe cassette. The Forward component was designed to maintain the correct expression and function of the target gene after forward integration of the FoRe donor into an intron target site or conditional inversion of the reverse-integrated donor by Cre recombinase. It contains the downstream intron sequence of the target site, including the splicing acceptor, as well as the full downstream coding sequence of the target gene, followed by a *tdTomato* reporter gene, separated by an in-frame 2A peptide coding sequence, to label the expression of the positive (functionally normal) allele. An *SV40 poly-A* signal (*pA*) was inserted after these coding sequences and used to terminate transcription (Fig. S1). The Reverse component was designed to disrupt the function of the target gene after reverse integration into the target locus or conditional inversion of the forward-integrated donor by Cre recombinase, and contains the downstream intron sequence of the target site, including the splicing acceptor, and only part of downstream in-frame coding sequence of the target gene, followed by an *EGFP* reporter gene, separated by an in-frame 2A peptide coding sequence, and a *BGH pA* to label the expression of the negative (mutant/defective) allele (Fig. S1). The Reverse component (including the *lox71* site) was cloned downstream to the Forward component (including the *lox66* site) in an opposite direction to form the FoRe cassette (Fig. S1) (Albert et al., 1995; Araki et al., 2002; Araki et al., 2006; Carney and Mosimann, 2018). Cre-induced recombination between the opposite *lox66* and *lox71* sites would result in the inversion of the FoRe cassette as well as generation of a wild-type *loxP* site and a mutant *lox72* site flanking in an opposite orientation (Fig. S1), leading to either conditional disruption of the target gene from the forward-integrated KI allele or conditional rescue of the target gene from the reverse-integrated KI allele.

When a FoRe donor was co-injected into one-cell stage zebrafish embryos together with the CRISPR/Cas system, the donor could be linearized and integrated into the intron target site in two directions. Upon forward integration, the KI allele serves as a positive conditional allele, where the Forward component will be transcribed and the coding sequence will be spliced with the upstream endogenous transcript, ensuring normal expression and function of the target gene as well as its fluorescent labelling by the *tdTomato* reporter, under normal conditions. In the presence of Cre recombinase, the FoRe cassette could be inverted and the defective Reverse component expressed, leading to disruption of the target gene as well as fluorescent labelling switch from tdTomato to EGFP (Fig. S1). On the other hand, through integration in the reverse direction, the KI allele would serve as a negative conditional allele. Under normal conditions, the Reverse component would be transcribed, together with its upstream plasmid backbone sequence, and theoretically correct splicing would lead to production of a truncated/defective protein and disruption of target gene function, as well as labelling of the negative conditional allele by the *EGFP* reporter. Restoration of target gene function could be achieved by Cre-dependent inversion of the reverse-integrated FoRe cassette in this negative conditional allele and subsequent correct expression of the Forward component, thus achieving conditional gene rescue (Fig. S1). By this strategy, positive and negative conditional allele pairs could be generated through forward and reverse KI, respectively, contemporarily within one experiment and using only one donor vector. Furthermore, tdTomato-labeling of functionally normal positive alleles and EGFP-labeling of functionally defective negative alleles could also be achieved at the same time, which can easily be used to trace and distinguish individual live embryos bearing different genotypes by fluorescent signals prior to the emergence of any visible phenotype.

### Efficient generation and characterization of the *sox10* positive conditional FoRe alleles

**Figure 1:**
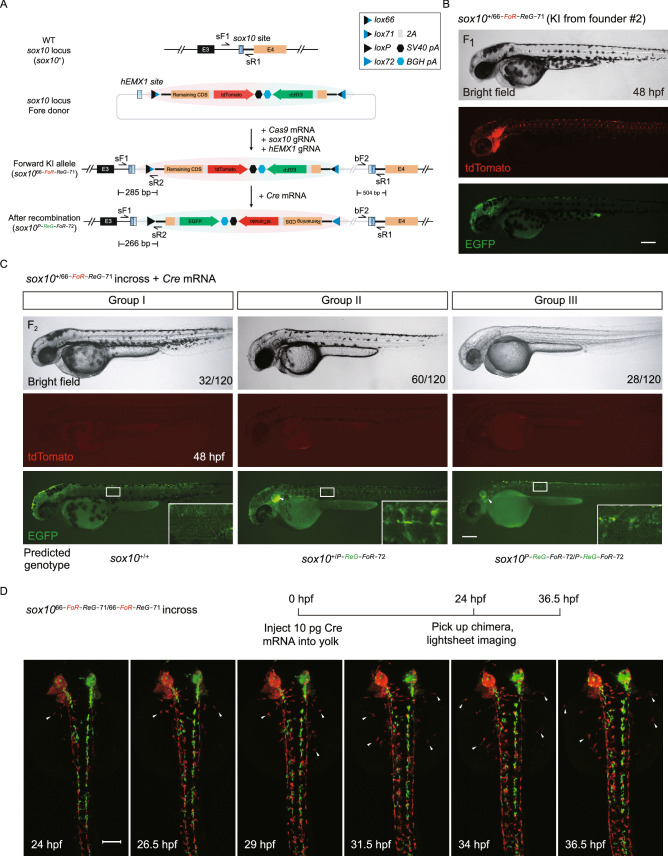
Generation and evaluation of fluorescent reporter-tagged conditional knockout alleles at the zebrafish ***sox10*** locus and mosaic tracing analysis of ***sox10*** expressing cells. (A) Schematic diagram of the KI strategy of *sox10* FoRe donor consisting of two components in opposite orientations (highlighted by red shadow for the Forward component for maintaining the function of *sox10*, and green shadow for the Reverse component for disrupting the function of *sox10*). The *sox10* CRISPR/Cas target site is shown as a dark blue box, and the *hEMX1* target site is shown as a light blue box. (B) Images of a 48 hpf F_1_ embryo from germline transmission screening of the *sox10* FoRe donor KI founder. Scale bar, 200 μm. (C) Phenotype analysis of the 48 hpf F_2_ embryos from the incross of *sox10*^*+/66-PoR-ReG−71*^ heterozygotes (derived from F_0_ #2) after the injection of 50 pg *Cre* mRNA at the one-cell stage. The white arrowheads indicate otic vesicles. Detailed sox10 expression in the trunk region can be seen under higher magnification of the boxed areas. Scale bar, 200 μm. (D) Serial lightsheet images of a *sox10* mosaic embryo from incross of *sox10*^*66-FoR-ReG−71/66-FoR-ReG−71*^ homozygotes after vegetal pole injection of 10 pg *Cre* mRNA and recorded from 24 hpf to 36.5 hpf. Some of the tdTomato-positive neural crest cells could migrate to the two sides of the body (as indicated by the white arrowheads), while all of the EGFP-positive cells remained in the middle. Scale bar, 100 μm

We first evaluated our FoRe donor strategy at the zebrafish *sox10* locus (Fig. 1A), which is primarily expressed in otic vesicle cells and neural crest cells (Dutton et al., 2001a, b; Geng et al., 2013). We identified a highly efficient CRISPR/Cas9 target site at the third intron of the *sox10* locus (Fig. S2A; Table S1). After co-injection of the *sox10* FoRe donor with *Cas9* mRNA, *sox10* gRNA, and *hEMX1* gRNA into one-cell stage embryos, 40.9% (38/93) of the embryos showed tdTomato fluorescent signal in the otic vesicle and pigment cells, derivatives of neural crest cells, at 36 hpf (hours post-fertilization) indicating efficient forward insertion of the donor vector (Fig. S2B; Table S2). Unfortunately, only a few (3/93) embryos showed limited EGFP signal from the same experiment, suggesting either inefficient insertion in the reverse direction or inefficient/incorrect transcription and/or splicing of the Reverse component. Founder embryos showing broad red fluorescence patterns (29/38) (Fig. S2B) were raised to adulthood and screened for germline transmission by outcross with wild-type zebrafish. 57.1% (4/7) of these founders gave rise to offspring showing correct red fluorescent signals, recapitulating the expression pattern of *sox10* in otic vesicle and pigment cells, as well as trunk neural crest cells (Figs. 1B and S2C; Table S3). Junction PCR results using pairs of primers spanning the 5′ junction and 3′ junction, as well as further sequencing results, confirmed expected NHEJ-mediated forward KI events at the *sox10* locus in the embryos positive for red fluorescent signals (Fig. S2D). The offspring from founder #2 were used for the subsequent experiments and the corresponding forward positive conditional FoRe allele was named *sox10*^*66-FoR-ReG−71*^. Unfortunately, we failed to obtain any heritable green fluorescent labeling of *sox10* expression, indicative of reverse insertion, from germline screening of 25 founder fish derived from either EGFP-positive or EGFP-negative embryos. We injected *Cre* mRNA into one-cell stage F_1_ embryos from founder #2, and observed the expected switch of the fluorescent signals from red to green (Fig. S2C), indicating successful inversion of the FoRe cassette in response to the Cre recombinase. The resulting inverted mutant allele was named *sox10*^*P-ReG-FoR−72*^. 5′ junction PCR and sequencing results further confirmed Cre-mediated recombination of this *sox10* positive conditional FoRe allele in the F_1_ embryos (Fig. S2E and data not shown). Note that the 3′ junction will not change after Cre-mediated recombination (Fig. 1A).

To further test the conditional effect of the *sox10*^*66-FoR-ReG−71*^ allele, the F_2_ progeny from incross of *sox10*^+/*66-FoR-ReG−71*^ heterozygotes were injected with 50 pg *Cre* mRNA per embryo at the one-cell stage. All the injected embryos showed little tdTomato signal at 48 hpf, suggesting efficient switch of the fluorescent reporter (Fig. 1C). Among these embryos, 26.7% (32/120) showed normal pigmentation without any fluorescent signal at 48 hpf (Fig. 1C, Group I), indicating these embryos were most likely to be *sox10*^+/+^. 50% (60/120) of the injected embryos showed EGFP fluorescent signal and slightly fewer pigment cells (Figs. 1C and S3A, Group II), indicating these embryos are likely to be heterozygous for the inverted FoRe allele, i.e., *sox10*^+/*P-ReG-FoR−72*^. 23.3% (28/120) of the injected embryos showed extensive loss of pigmentation (Figs. 1C and S3A, Group III), and these embryos were all positive for EGFP expression, which suggested that they were most likely homozygous for the inverted FoRe allele, i.e., *sox10*^*P-ReG-FoR−72*/*P-ReG-FoR−72*^. PCR genotyping results confirmed the expected correlation between the genotype and the phenotype in these three groups of embryos (Fig. S3B). To evaluate the effect of the *sox10* KI allele in its defective state after Cre-induced recombination at the molecular level, we performed RT-PCR experiments to measure the expression of *sox10* on the embryos from incross of the *sox10*^+/*66-FoR-ReG−71*^ heterozygotes with *Cre* mRNA injection. The results showed significant reduction of functionally normal *sox10* transcripts in the homozygous *sox10*^*P-ReG-FoR−72*/*P-ReG-FoR−72*^ mutant embryos, as expected (Fig. S3C), indicating highly efficient mutagenesis by the *sox10*^*P-ReG-FoR−72*^ allele. These results together demonstrated that our FoRe strategy could efficiently generate fluorescent reporter-tagged positive conditional KI alleles and successfully achieve CKO coupled with simultaneous fluorescent allele-label switching.

### Mosaic analysis and tracing of *sox10-*expressing cells facilitated by the FoRe KI allele

Since cells with different genotypes could be labeled with different fluorescent reporters, our FoRe KI strategy provides a unique opportunity for real-time mosaic analysis as well as cell fate tracing in live embryos. We injected 10 pg *Cre* mRNA into the vegetal pole of the progeny from incross of *sox10*^*66-FoR-ReG−71*/*66-FoR-ReG−71*^ homozygotes, then screened chimeras showing both red and green fluorescent signals at 24 hpf and traced neural crest cell fate for 12.5 hours by live imaging under a lightsheet microscope. The results showed that neural crest cells showing only tdTomato expression displayed the ability to migrate to both the left and right sides of the embryo body, whereas cells with only EGFP labeling were clearly lagging behind with most of the cells staying around the midline (Fig. 1D; Movie S1), indicating that neural crest cells homozygous for mutant *sox10*, as revealed by the EGFP signal, are defective for lateral migration, which is consistent with previous reports (Kelsh and Eisen, 2000; Dutton et al., 2001b). This observation demonstrated the advantage of the capability of our FoRe system for tracing and comparing functionality of cell lineages with different genetic backgrounds in parallel within one individual.

### Application of the FoRe strategy at the zebrafish *isl1* locus

Since we did not obtain stable reverse integration of the FoRe donor at *sox10* locus, we next evaluated our strategy at a second locus *isl1*, encoding a DNA-binding transcription factor which is involved in multiple developmental processes, including atrial cardiac muscle cell differentiation, neuron differentiation and pancreas development (Sirbu et al., 2008; Witzel et al., 2012; Wilfinger et al., 2013; Caputo et al., 2015; Witzel et al., 2017). We identified a highly efficient CRISPR/Cas9 target site at the third intron of *isl1* (Fig. S4A; Table S1). After co-injection of an *isl1* FoRe donor, *Cas9* mRNA, *isl1* gRNA, and *hEMX1* gRNA into one-cell stage zebrafish embryos (Fig. S4B), about 16.5% (13/79) of the injected embryos showed tdTomato expression (Fig. S4C; Table S2), but no embryos expressing EGFP were observed, again indicating efficient forward but not reverse insertion. The potential founders showing tdTomato expression were raised to adulthood and germline screening was conducted by outcross with wild-type zebrafish. 60% (3/5) of the founders produced offspring showing correct tdTomato expression in eyes and trunk motor neurons, indicating stable inheritance of the positive allele derived from forward KI of the FoRe donor (Fig. S4D and S4E; Table S4). Junction PCR as well as sequencing results confirmed correct NHEJ-mediated forward KI events at the *isl1* locus (Fig. S4F). The offspring from founder #2 were used for the following experiments and the corresponding positive conditional FoRe allele was named as *isl1*^*66-FoR-ReG−71*^. We injected 10 pg of *Cre* mRNA into one-cell stage F_1_ embryos from the outcross of this founder, and observed the expected switch of the fluorescent signals from red to green in some cells (Fig. S4E), and the knockout allele derived from Cre-induced inversion was named as *isl1*^*P-ReG-FoR−72*^. PCR and sequencing results further confirmed Cre-induced recombination in the injected embryos with EGFP expression (Fig. S4G).

To evaluate the CKO effect of our flipping donor strategy at the *isl1* locus, we injected 50 pg *Cre* mRNA into the animal pole of F_2_ progeny from incross of the *isl1*^*+/66-FoR-ReG−71*^ F_1_ heterozygotes at the one-cell stage. All of the injected embryos showed little tdTomato signal, suggesting efficient inversion of the FoRe cassette (Fig. S5A). Among these embryos, 23.6% (22/93) exhibited defective phenotypes such as reduced head and body size, abnormal heart, and curved body axis at 48 hpf, and all these defective embryos were positive for EGFP expression (Fig. S5A, Group III), indicating that these embryos were most likely *isl1*^*P-ReG-FoR−72/P-ReG-FoR−72*^ homozygous mutants. 53.8% (50/93) of the injected embryos developed normally and also showed EGFP fluorescent signal, suggesting these embryos may be *isl1*^*+/P-ReG-FoR−72*^ heterozygous (Fig. S5A, Group II). 22.6% (21/93) of the injected embryos showed normal embryogenesis without any fluorescent signal, indicating that they were likely *isl1*^*+/+*^ wild-type embryos (Fig. S5A, Group I). PCR genotyping results further confirmed the correlation between genotype and phenotype in each group, as expected (Fig. S5B). To measure the expression of the *isl1*^*66-FoR-ReG−71*^ allele in its defective state after Cre-induced inversion, we performed RT-PCR experiments on the mutant embryos from incross of the *isl1*^*+/66-FoR-ReG−71*^ heterozygotes with *Cre* mRNA injection. The results showed that little *isl1* transcript was detected in the mutant embryos (Fig. S5C), indicating highly efficient knockout effect of the *isl1*^*P-ReG-FoR−72*^ allele derived from the inversion of the* isl1*^*66-FoR-ReG−71*^ allele. Taken together, these results demonstrated the generality and high efficiency of our FoRe KI strategy for generating positive conditional alleles together with genotype labeling effect through forward insertion of the FoRe donor. However, we failed to effectively isolate negative conditional KI alleles which should derive from reverse integration of the FoRe donor (to allow for conditional rescue effect) through pre-selection of founder embryos by EGFP expression, indicating that the design of our FoRe strategy needed further improvement.

### Improvement of our FoRe strategy to achieve contemporary generation of positive and negative conditional alleles with fluorescent allele tagging effect

**Figure 2:**
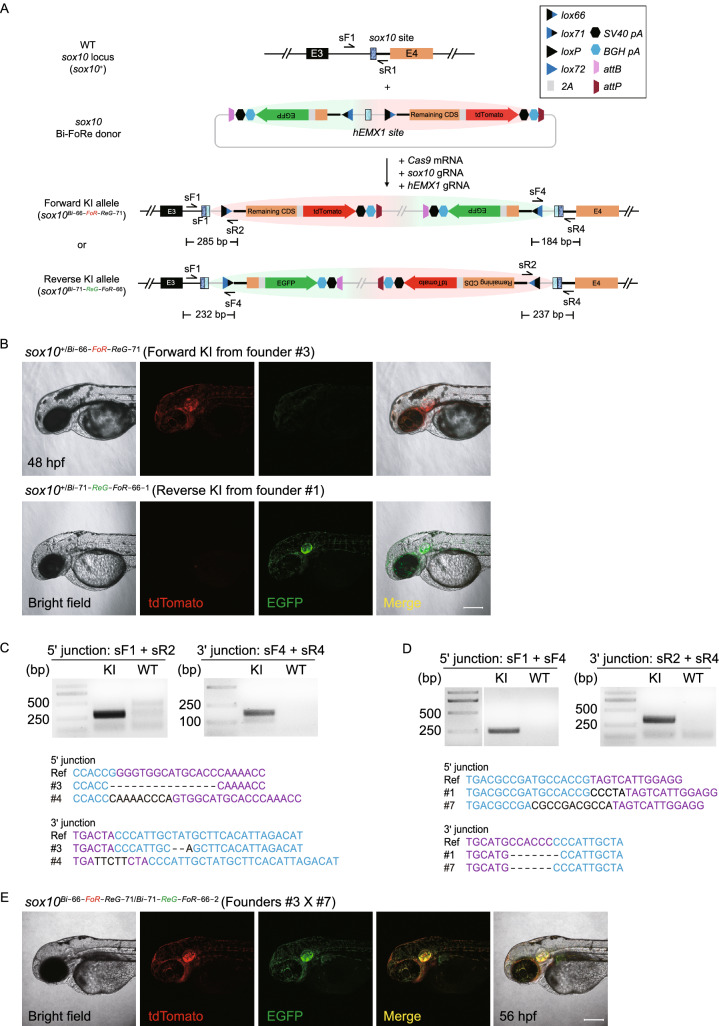
Generation of positive and negative conditional allele pairs at the ***sox10*** locus through the Bi-FoRe strategy. (A) Schematic diagram of the improved KI strategy based on the bidirectional multi-purpose Bi-FoRe donor consisting of two functional components. The Forward component (highlighted by red shadow) is designed to maintain the function of the *sox10* gene, and the Reverse component (highlighted by green shadow) is designed to disrupt the *sox10* function. The *sox10* CRISPR/Cas target site is shown in dark blue, and the *hEMX1* target site is shown in light blue, located in the middle of the two functional components in the donor, to facilitate unbiased identification of both forward and reverse integrations of the donor. (B) Z-stack confocal images of a 48 hpf *sox10*^*+/Bi−66-FoR-ReG−71*^ F_1_ embryo from outcross of founder #3 and a 48 hpf *sox10*^*+/Bi−71-ReG-FoR−66−1*^ F_1_ embryo from outcross of founder #1, respectively. Scale bar, 200 μm. (C) Junction PCR and direct sequencing results of F_1_ progeny showing tdTomato expression from outcross of F_0_ #3 or #4, demonstrating forward insertion of the* sox10* Bi-FoRe donor. KI: pooled genomic DNA template of F_1_ embryos from outcross of F_0_ #3. WT: pooled genomic DNA template of wild-type embryos. (D) Junction PCR and direct sequencing results of F_1_ progeny showing EGFP expression from outcross of F_0_ #1 or #7, demonstrating reverse insertion of the *sox10* Bi-FoRe donor. KI: pooled genomic DNA template of the F_1_ embryos from outcross of F_0_ #1. WT: pooled genomic DNA template of wild-type embryos. (E) Z-stack confocal images of a 56 hpf *sox10*^*Bi−66-FoR-ReG−71/Bi−71-ReG-FoR−66−2*^ F_1_ embryo from the cross between founders #3 and #7, showing overlapping expression of tdTomato and EGFP. Scale bar, 200 μm

We noticed that EGFP-positive embryos (representing reverse insertion) were much less frequent than those expressing tdTomato (representing forward insertion) after injection of the FoRe donor KI system at both *sox10* and *isl1* loci. The major difference between forward insertion and reverse insertion with regard to the reporter gene is its relative position and distance to the insertion site in the zebrafish genome, or more precisely, the relationship with its upstream endogenous genomic sequence. Almost no extra sequence is placed before the *tdTomato* reporter besides the essential target gene sequence in the case of forward insertion; in contrast, in reverse insertion, there is a long exogenous bacterial plasmid backbone sequence (approximately 2,600 bp) lying between the negative Reverse component and its upstream genomic insertion site. We suspect that this exogenous sequence may contain cryptic splicing acceptor(s) which might disturb the correct and/or efficient splicing of the transcript from the Negative component into the upstream endogenous portion of the transcript, therefore leading to reduced EGFP fluorescent signal. To solve this potential problem, we improved the design of our FoRe donor by moving the Reverse component containing two *pA* signals to upstream of the *hEMX1* site, so that this donor linearization site was placed between the two functional components (Fig. 2A). In this manner, the relative position of the *EGFP* reporter within the targeted genome after reverse integration will be comparable with the position of the *tdTomato* reporter after forward integration, eliminating potential interference from the plasmid backbone (Fig. 2A). We call this improved vector Bi-FoRe (bidirectional FoRe) donor. Additionally, physical elimination of the plasmid backbone could be further considered, by using minicircle DNA technology for the construction of the donor vector, which contains *attB* and *attP* sites flanking the plasmid backbone that could be removed either *in vitro* or *in vivo* through phiC31-induced recombination (Kay et al., 2010; Suzuki et al., 2016) (Fig. 2A).

### Generation of bidirectional and conditional Bi-FoRe KI allele pairs at the *sox10* locus

We evaluated the Bi-FoRe strategy at the* sox10* locus. After co-injection of the *sox10* Bi-FoRe donor with *Cas9* mRNA, *sox10* gRNA, and *hEMX1* gRNA into one-cell stage zebrafish embryos, 20.7% (28/135) of the embryos showed tdTomato signal and 42.2% (57/135) showed EGFP signal (Table S2). Interestingly, 16 embryos exhibited mosaic expression of both tdTomato and EGFP signals, indicating that both forward and reverse integrations happened efficiently in the same embryo. Potential founders showing broad fluorescent patterns were raised to adulthood and germline transmission screening was conducted by outcrossing with wild-type zebrafish. Three out of 18 founders (#3, #4, #9) produced offspring showing only tdTomato expression (Fig. 2B; Table S5), with normal phenotype and identical fluorescence pattern to the *sox10*^*+/66-FoR-ReG−71*^ F_1_ embryos carrying the previous FoRe donor KI allele, as expected. The offspring from founder #3 were used for the following experiments and the corresponding forward positive conditional Bi-FoRe KI allele was named as *sox10*^*Bi−66-FoR-ReG−71*^. Four out of the same 18 founders (#1, #2, #7, #8) produced offspring showing only EGFP expression (Fig. 2B; Table S6), the offspring from founders #1 and #7 were used for the subsequent experiments and the corresponding reverse negative conditional Bi-FoRe KI alleles were named as *sox10*^*Bi−71-ReG-FoR−66−1*^ and *sox10*^*Bi−71-ReG-FoR−66−2*^, respectively. Interestingly, two founders (#5, #6) showed germline transmission of both tdTomato and EGFP expression, demonstrating high KI as well as germline transmission efficiency of our new strategy (Tables S5 and S6). As expected, junction PCR and sequencing results confirmed correct NHEJ-mediated KI events of these alleles at the *sox10* locus (Fig. 2C and 2D). Efficient germline transmission of both forward and reverse KI events at the *sox10* locus proved the feasibility of our Bi-FoRe donor strategy as a time-saving one-step efficient method to generate multi-purpose labeled genome modifications in pairs. On one hand, *sox10*^*Bi−66-FoR-ReG−71*^ and *sox10*^*Bi−71-ReG-FoR−66−1*^ (and *sox10*^*Bi−71-ReG-FoR−66−2*^) can be considered as a pair of stable positive (or functionally normal) and negative (or mutant/defective) alleles tagged with different fluorescent reporters; on the other hand, they are also a pair of conditional knockout and conditional rescue alleles, each bearing different fluorescent reporters.

### Characterization and application of the phenotype and allele-labeling effect of the *sox10* Bi-FoRe KI allele pairs

**Figure 3:**
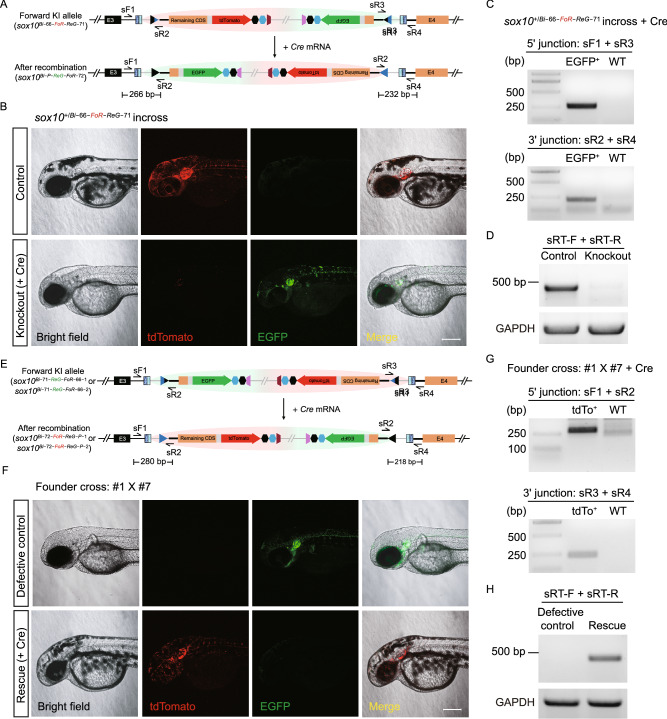
Evaluation of conditional manipulation of paired positive and negative conditional Bi-FoRe KI alleles at the ***sox10*** locus. (A) Schematic diagram of the positive conditional allele (Forward KI allele) *sox10*^*Bi−66-FoR-ReG−71*^ before and after Cre-mediated recombination. (B) Z-stack confocal images of F_2_ embryos obtained from incross of F_1_ *sox10*^*+/Bi−66-FoR-ReG−71*^ heterozygotes. Upper panel: An embryo without *Cre* mRNA injection, showing tdTomato expression and normal pigmentation. Lower panel: An embryo after *Cre* mRNA injection, showing EGFP expression and defects in pigmentation, resembling sox10 mutant phenotype. Scale bar, 200 μm. (C) Junction PCR results of the F_2_ embryos from incross of *sox10*^*+/Bi−66-FoR-ReG−71*^ after* Cre* mRNA injection. EGFP^+^: pooled genomic DNA template of the F_2_ progeny showing EGFP expression after *Cre* mRNA injection. WT: pooled genomic DNA template of wild-type embryos. (D) RT-PCR results using the cDNA of the F_2_ embryos from B with (Knockout) or without (Control) *Cre* mRNA injection. (E) Schematic diagram of the negative conditional alleles (Reverse KI allele) *sox10*^*Bi−71-ReG-FoR−66−1*^ and *sox10*^*Bi−71-ReG-FoR−2*^ before and after Cre-mediated recombination. (F) Z-stack confocal images of the embryos obtained from a cross of F_0_ #1 bearing the *sox10*^*Bi−71-ReG-FoR−66−1*^ allele with #7 bearing the *sox10*^*Bi−71-ReG-FoR−66−2*^ allele. Upper panel: F_1_ embryo without *Cre* mRNA injection, showing EGFP expression as well as defects in pigmentation, resembling *sox10* mutant phenotype. Lower panel: F_1_ embryo with *Cre* mRNA injection, showing tdTomato expression and recovery of pigmentation. Scale bar, 200 μm. (G) Junction PCR results of the F_1_ embryos from the cross of F_0_ #1 with #7 after* Cre* mRNA injection. tdTo^+^: pooled genomic DNA template from the F_1_ progeny showing tdTomato expression after *Cre* mRNA injection. WT: pooled genomic DNA template of wild-type embryos. (H) RT-PCR results using the cDNA of the embryos from panel F with (Rescue) or without (Defective control) *Cre* mRNA injection. The location of RT-PCR primers in D and H is indicated in Fig. S3C. The expected size of the band is 467 bp

We further characterized and compared the phenotype as well as expression of the positive *sox10*^*Bi−66-FoR-ReG−71*^ and negative *sox10*^*Bi−71-ReG-FoR−66−1*^ (or *sox10*^*Bi−71-ReG-FoR−66−2*^) alleles under normal conditions (without introduction of Cre recombinase), separately or in pairs. To evaluate whether loss-of-function phenotype and correct reporter gene expression can be detected in the zebrafish carrying the negative *sox10* Bi-FoRe KI alleles, we crossed founder #1 with founder #7 and obtained *sox10*^*+/Bi−71-ReG-FoR−66−1*^ and *sox10*^*+/Bi−71-ReG-FoR−66−2*^ heterozygotes, as well as *sox10*^*Bi−71-ReG-FoR−66−1/Bi−71-ReG-FoR−66−2*^ compound heterozygous progeny. In contrast to the positive *sox10*^*Bi−66-FoR-ReG−71*^ allele, the embryos derived from the founders bearing the negative* sox10*^*Bi−71-ReG-FoR−66−1*^ or *sox10*^*Bi−71-ReG-FoR−66−2*^ allele showed only green but not red fluorescent signals (Fig. 2B). In addition, some of the green-fluorescent embryos showed partial reduction of pigmentation, while others were completely devoid of pigmentation in their trunk region at 48 hpf (Fig. 3F, upper panels), which largely recapitulated the expected phenotype of sox10 mutation and suggests that they correspond to *sox10*^*+/Bi−71-ReG-FoR−66−1*^ or *sox10*^*+/Bi−71-ReG-FoR−66−2*^ heterozygotes and *sox10*^*Bi−71-ReG-FoR−66−1/Bi−71-ReG-FoR−66−2*^ compound heterozygotes, respectively. This result indicates that either *sox10*^*Bi−71-ReG-FoR−66−1*^ or *sox10*^*Bi−71-ReG-FoR−66−2*^ allele could efficiently disrupt the function of the *sox10* gene. To achieve stable and homogeneous expression tagging of both positive/normal and negative/defective *sox10* alleles by tdTomato and EGFP within the same embryo, respectively, we crossed founders #3 and #7, and obtained F_1_ embryos showing both red and green fluorescent signals. These *sox10*^*Bi−66-FoR-ReG−71/Bi−71-ReG-FoR−66−2*^ embryos showed overlapping expression patterns for the two fluorescent reporters as well as partial reduction of pigmentation similar to the *sox10*^*+/Bi−71-ReG-FoR−66−2*^ heterozygous F_1_ embryos, as expected, again indicating that both the tdTomato reporter in the positive *sox10*^*Bi−66-FoR-ReG−71*^ allele and the EGFP reporter in the negative *sox10*^*Bi−71-ReG-FoR−66−2*^ allele could faithfully label the expression of the *sox10* locus, and furthermore, the positive *sox10*^*Bi−66-FoR-ReG−71*^ allele and negative *sox10*^*Bi−71-ReG-FoR−66−2*^ allele could successfully recapitulate normal and defective *sox10* function, respectively (Fig. 2E).

To evaluate the potential utility of the double labeling of the positive and negative *sox10* allele pair to reveal cellular genotypes at early embryonic stages before the appearance of visible phenotype, we further analyzed the timing of phenotype and reporter gene expression in embryos obtained from the outcross of *sox10*^*+/Bi−66-FoR-ReG−71*^ with wild type fish and those from the cross between #1 and #7 founders, at early developmental stages. Both red and green fluorescent signals could be detected separately in the corresponding embryos before 14 hpf by lightsheet microscopy (data not shown) and were easily observable under general compound fluorescence microscopy at 21 hpf, displaying similar patterns, prior to the emergence of melanocytes (Fig. S6A), while phenotypic difference in pigmentation between the different genotypes (as revealed by fluorescent signals and confirmed by PCR genotyping) was not visible until 28 hpf (Fig. S6B and S6C), consistent with the previous report (Kelsh and Eisen, 2000). Taken together, these results demonstrated that different fluorescent labeling of the positive/normal and negative/defective alleles by our new Bi-FoRe strategy will aid early *in vivo* discrimination of different genotypes in embryos before or even without the requirements for the onset of any visible phenotype, especially for large numbers of embryos, which is critical for early and accurate dissection of target gene function and molecular mechanisms.

### Evaluation of the conditional manipulation of the *sox10* Bi-FoRe KI allele pairs

Next, to evaluate the conditional knockout effect of the floxed positive *sox10*^*Bi−66-FoR-ReG−71*^ allele, the F_2_ progeny from the incross of *sox10*^*+/Bi−66-FoR-ReG−71*^ heterozygotes were injected with 50 pg *Cre* mRNA per embryo at the one-cell stage (Fig. 3A and 3B). While the un-injected control embryos showed only red fluorescent signals, most injected embryos exhibited only green fluorescent signal, indicating highly efficient switch of reporter gene expression from *tdTomato* to *EGFP*, and therefore highly efficient conversion of the functional *sox10*^*Bi−66-FoR-ReG−71*^ allele into the defective* sox10*^*Bi-P-ReG-FoR−72*^ allele. In addition, all EGFP-positive embryos exhibited either partial or complete loss of pigmentation, suggesting they are *sox10*^*+/Bi-P-ReG-FoR−72*^ heterozygotes and *sox10*^*Bi-P-ReG-FoR−72/Bi-P-ReG-FoR−72*^ homozygous mutants, respectively (Fig. 3B). Junction PCR results confirmed the expected Cre-mediated recombination events at the *sox10* locus (Fig. 3C), and genotyping results of the embryos devoid of any pigmentation further confirmed that these embryos were indeed homozygous of the Bi-FoRe KI allele *sox10*^*Bi−66-FoR-ReG−71*^ before *Cre* mRNA injection (Fig. S7A). RT-PCR experiments detected little normal *sox10* transcript in the embryos devoid of any pigmentation after *Cre* mRNA injection (Fig. 3D), confirming that the *sox10*^*Bi-P-ReG-FoR−72*^ is an effective knockout allele.

Compared with the original FoRe strategy, a unique advantage of our Bi-FoRe strategy is the efficient generation of stable negative alleles by germline transmission of the reverse-integrated Bi-FoRe donor. These alleles are functionally defective for the *sox10* gene and express an *EGFP* reporter under normal conditions (i.e., without Cre recombinase), and exhibit conditional rescue as well as fluorescent reporter switch in the presence of Cre recombinase, i.e., they could be converted into functionally normal *sox10* alleles, coupled with a switch of the reporter gene expression from *EGFP* to *tdTomato*, after Cre-induced inversion of the Bi-FoRe cassette. To evaluate whether conditional rescue of gene expression and function can be achieved in zebrafish carrying the negative *sox10*^*Bi−71-ReG-FoR−66−1*^ and *sox10*^*Bi−71-ReG-FoR−66−2*^ alleles, we crossed the corresponding founder #1 with founder #7 and injected *Cre* mRNA into some of the one-cell stage progeny (Fig. 3E and 3F). While the un-injected control embryos showed only green fluorescent signal, injected embryos exhibited mostly red fluorescent signal, indicating highly efficient switch from *EGFP* to *tdTomato* reporter gene expression, as well as highly efficient conversion of the *sox10*^*Bi−71-ReG-FoR−66−1*^ and *sox10*^*Bi−71-ReG-FoR−66−2*^ alleles into *sox10*^*Bi−72-FoR-ReG-P−1*^ and *sox10*^*Bi−72-FoR-ReG-P−2*^, respectively (Fig. 3F). Furthermore, all of the tdTomato-expressing embryos displayed complete recovery of body pigmentation, indistinguishable from their wild-type siblings (i.e., those embryos without any fluorescent signal), confirming successful and efficient rescue of *sox10* function (Fig. 3F). Junction PCR results further confirmed expected Cre-mediated recombination events at the *sox10* locus (Fig. 3G). We randomly selected seven injected embryos expressing only tdTomato for genotyping, and results showed that two of these embryos did not contain the wild-type allele, indicating they harbored Bi-FoRe KI alleles from both founder #1 and #7 (Fig. S7B), indicating efficient rescue of the original mutants by the Cre recombinase. We also performed RT-PCR experiments on the un-injected mutant embryos and *Cre*-injected rescue embryos from Fig. 3F, and the results showed that *sox10* expression was largely recovered in the *Cre* mRNA injected embryos compared with the original un-injected embryos (Fig. 3H). Taken together, our Bi-FoRe KI strategy provides a simple, versatile and efficient method to simultaneously generate both positive and negative conditional alleles to be used for CKO and conditional gene rescue analyses in conjunction with allele-labeling effect using a single donor vector within a single experiment.

### Elimination of the plasmid backbone by phiC31-mediated *attB/attP* recombination *in vivo* or *in vitro*

Integration of a plasmid backbone into the eukaryotic genome might induce DNA methylation and silencing of transcription (Suzuki et al., 2016), interfering with the function and inheritance of the KI alleles. We tested two approaches to eliminate the undesirable influence of the bacterial backbone sequence, first by removing the backbone using flanking *attB* and *attP* sites through *in vivo* phiC31-induced excision in the KI embryos, and secondly by replacing the donor vector with a backbone-free minicircle plasmid for the KI experiments. To test the first approach, we injected* phiC31* mRNA into offspring from the outcross of *sox10*^*+/Bi−66-FoR-ReG−71*^ or *sox10*^*+/Bi−71-ReG-FoR−66−1*^. Recombination between *attB* site and *attP *site via phiC31 results in the removal of their flanked sequence and production of an* attR* site and an* attL* site (Lu et al., 2011; Carney and Mosimann, 2018), and the alleles without backbone sequence were referred to as *sox10*^*Mini−66-FoR-ReG−71*^ (Fig. S8A) and *sox10*^*Mini−71-ReG-FoR−66−1*^, respectively. PCR and sequencing results confirmed the phiC31-mediated recombination events in the injected embryos (Fig. S8B and data not shown), and the expression pattern of the fluorescent reporters was comparable with the un-injected embryos (Fig. S8C). These results demonstrated the feasibility of removing the donor backbone by using the phiC31 and *attB/attP* system *in vivo*.

**Figure 4:**
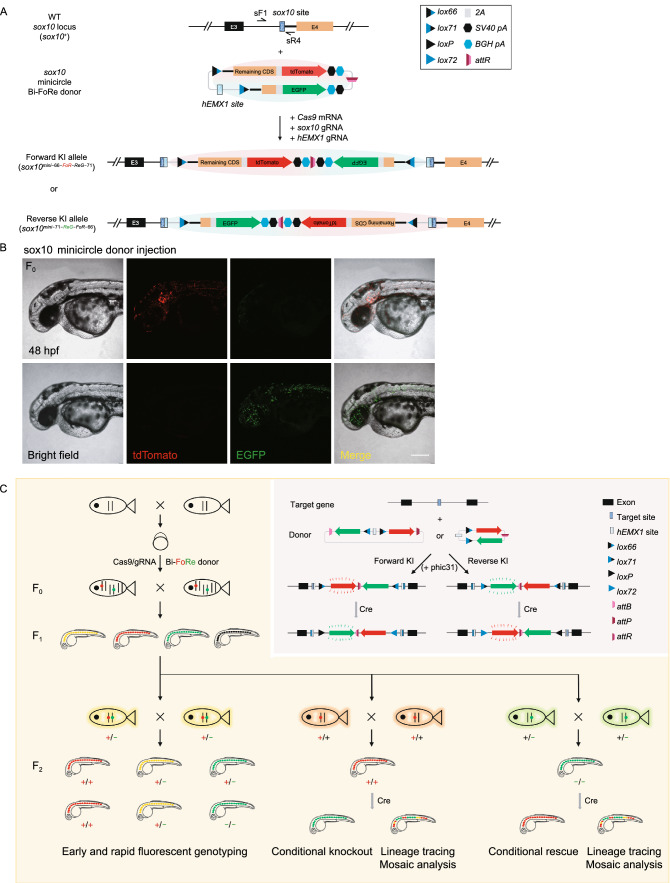
Generation of conditional ***sox10*** KI alleles using minicircle donor and summary of our bidirectional KI strategy. (A) Schematic diagram of the KI strategy based on the minicircle Bi-FoRe donor derived from *in vitro* backbone elimination. (B) Representative z-stack confocal images of two 48 hpf founder embryos after injection of the *sox10* minicircle Bi-FoRe donor vector together with the CRISPR/Cas9 system, showing red (representing forward insertion) and green (representing reverse insertion) fluorescent signals, respectively. Scale bar, 200 μm. (C) A graphical summary of the principle and applications of our Bi-FoRe KI strategy

*In vitro* removal of the plasmid backbone before introduction of the donor vector into the embryos through the minicircle system is an alternative approach to minimize the un-wanted influence from the plasmid vector, and furthermore, the relatively smaller size of the minicircle donor vector may improve the efficiency of targeted KI integration. To validate this assumption, we generated a *sox10* minicircle Bi-FoRe donor and injected it along with* Cas9* mRNA, *sox10* gRNA, and *hEMX1* gRNA into one-cell stage zebrafish embryos, in equimolar amounts with the full length donor (Fig. 4A). 51.7% (76/147) and 29.9% (44/147) of the injected embryos showed tdTomato and EGFP fluorescent signal, respectively (Fig. 4B; Table S2). Of these, 25 embryos showed both tdTomato and EGFP expression. Therefore, the overall KI efficiency in minicircle donor-injected founder embryos increased to 64.6%, compared with 51.1% for the injection of the full length Bi-FoRe donor containing the full-length plasmid backbone (Table S2), suggesting that minicircle Bi-FoRe donor may be more efficient for generating NHEJ-mediated KI.

## Discussion

In this study, we developed an efficient flipping donor strategy based on dual-functional FoRe and Bi-FoRe donors to simultaneously generate positive and negative conditional alleles, coupled with fluorescent allele tagging/geno-tagging, through NHEJ-mediated targeted insertion facilitated by the CRISPR/Cas system. The expression of the FoRe and Bi-FoRe KI alleles could be revealed in live embryos by the fluorescent reporters fused in-frame with the coding sequence of the target gene, while the function of these alleles could be conditionally manipulated by Cre-induced inversion of the FoRe or Bi-FoRe cassette due to the recombination between the opposite-orientated *lox66* and *lox71* sites. Paired multifunctional KI alleles, enabling both fluorescent labeling of positive and negative alleles on one hand, and conditional knock out and conditional rescue on the other hand, could be generated efficiently and simultaneously through bidirectional targeted insertion of the same donor vector in one experiment, which could largely simplify the experimental process and save both time and effort compared with current existing methods (Figs. 4C and S9).

The feasibility of our FoRe strategy was demonstrated at the* sox10* and *isl1* loci in zebrafish. We first generated double fluorescent reporter-labeled and floxed positive KI alleles of *sox10* and* isl1* genes through forward insertions of the FoRe donors. We then successfully achieved CKO effect as well as fluorescent tag switching through Cre-induced inversion of the FoRe cassette from its positive state to its negative state. We also performed real-time mosaic analysis and cell fate tracing on different genotypes side-by-side within a single embryo derived from conditional manipulation of the *sox10* FoRe KI alleles. By this experiment, we observed a clear difference in the migratory capacity between cells normal and defective in *sox10* function (Fig. 1D; Movie S1), which demonstrated the power of our method for tissue/organ specific mosaic analysis of different genotypes. However, we failed to isolate the expected negative conditional alleles which should derive from the reverse insertion. Concerned that the long bacterial backbone might interfere with identification of reverse insertion events, we improved the design by locating the backbone sequence within the FoRe cassette between the Forward component and the Reverse component, so both components are equally close to the integration site within the target gene after insertion. We named this improved donor Bi-FoRe, and successfully generated both positive and negative Bi-FoRe KI alleles at the *sox10* locus through bidirectional integration of this new donor within a single KI experiment. Based on these allele pairs, we successfully achieved conditional knockout and conditional rescue in parallel, both coupled with switch of fluorescent reporter genes, from positive and negative conditional alleles at the *sox10* locus, respectively.

Similar to other organisms, KI in zebrafish could be achieved by either homology-dependent or -independent approaches (Zu et al., 2013; Auer et al., 2014; Shin et al., 2014; Li et al., 2015; Hoshijima et al., 2016; Sugimoto et al., 2017; Burg et al., 2018; Luo et al., 2018; Li et al., 2019; Li et al., 2020). Inclusion of homology arms in the donor allows for precise integration of the donor through HR, while dramatically compromising the KI efficiency. In contrast, donors without homology arms could incorporate into the target site through NHEJ-induced insertion, which is more efficient than HR-mediated KI, though may be less precise due to introduction of indels at the 5′- and 3′- junctions. Since small indels in introns are usually tolerated, targeting introns for donor KI through the NHEJ pathway could make best use of its high efficiency and at the same time bypass the risk of disrupting gene function by imprecision integration. More importantly, NHEJ-mediated KI allows us to simutaneously generate useful insertions occurring in both directions, each displaying different but complementary KI effects, while homology-directed integration is generally unidirectional and thus inappropriate for our Bi-FoRe strategy. Interestingly, germline transmission rate in this study was high at both *sox10* and *isl1* loci. We believe there are at least two further reasons accounting for the high KI efficiency, in addition to the benefits of NHEJ-mediated targeted insertion. One is that we have chosen Cas9/gRNA target sites showing high indel efficiency. The other is that we pre-selected fluorescence-positive founder (F_0_) embryos after injection of the targeting system and before rearing for germline transmission screening. The importance of pre-selection for the enrichment of F_0_ embryos bearing correct insertions has been demonstrated in our previous publication (Li et al., 2019)

One of the major improvements of our Bi-FoRe strategy is the successful utilization of insertions occurring in both directions, due to the special design of our donor vector. Bidirectional KI enables concurrent generation of positive and negative alleles, allowing for *in vivo* monitoring of gene expression and conditional manipulation of gene function. To our knowledge, this is the first method available for simple and efficient one-step generation of multi-purpose bidirectional genome modifications, including pairwise fluorescent allele labeling and pairwise conditional allele manipulation. At least two important aspects or applications could be derived from the success of reverse integration, in conjunction with the forward insertion. On one hand, concurrent generation and separate labeling of positive and negative allele pairs with different fluorescent reporter genes could allow for simple and early discrimination of live embryos bearing different genotypes before or without the requirements for the appearance of any visible phenotype, which is crucial for timely and accurate dissection of gene function and mechanisms. This is especially important for timely isolation of large numbers of embryos, which is usually required for genome-wide gene expression and function analyses, such as transcriptome analysis through RNA sequencing, either in bulk or at the single-cell level. For example, the fluorescent signal of our *sox10* Bi-FoRe KI embryos could be detected at 21 hpf, seven hours before appearance of visible phenotype (Fig. S6), while the initial phenotype of *sox10* mutants is undetectable until 28 hpf. Thus, fluorescent allele labeling provides a powerful tool to capture the correct developmental stage initiating target gene expression and the genotype of the embryos as early as possible, facilitating precise characterization and elucidation of the primary and fundamental molecular events regulated by the target gene.

On the other hand, concurrent pairwise generation of conditional alleles from forward and reverse integration provides a unique opportunity for comprehensive and reciprocal elucidation of gene functions as well as molecular mechanisms. Conditional rescue is an important complementary approach for precise and in-depth analysis of gene function in development, regeneration, and disease progression. Time- and tissue-specific gene function restoration helps confirm the results from gene knockouts and allows detailed dissection of gene functions in different cell lineages and biological stages (Ruehle et al., 2013; Santos et al., 2016; Flores et al., 2018). Utilization of both CKO and conditional rescue analyses of the same gene can largely facilitate the elucidation of cell autonomous or non-autonomous functions of the target gene in adjacent cells and tissues, identification of the effector cells/tissues in which expression of the target gene is both necessary and sufficient, as well as elaboration of gene functions within a specific time window (Santos et al., 2016; Flores et al., 2018). Moreover, time- or tissue-specific recovery of gene expression in embryonic or adult animal disease models can contribute to pathological and therapeutic studies of the affected genes, and thus reveal specific timings and target cells/tissues for gene therapy treatments intended for clinical application, which cannot be achieved by conditional knockouts (Guy et al., 2007; Mei et al., 2016). However, despite these uses, conditional rescues are not widely applied in the studies of gene functions, largely due to the relative complexity and extra effort required in allele construction. Our Bi-FoRe donor strategy provides an efficient method to establish both conditional knockout and conditional rescue alleles of the same gene at the same time within a single experiment, which greatly reduces the time and effort involved in donor construction and allele identification, thus opening new possibilities for detailed dissection of gene function in multiple dimensions.

In the Bi-FoRe KI alleles derived from the full length donor, in addition to the necessary functional sequences, a long bacterial plasmid backbone sequence was also inserted into the target locus. It has been reported that exogenous bacterial sequences introduced into the eukaryotic genome may cause alteration of the expression level of endogenous genes (Chen et al., 2001, 2003; Suzuki et al., 2016). To avoid potential deleterious consequences, we adopted the minicircle vector pTUBB3-MC to construct our Bi-FoRe KI donor (Kay et al., 2010; Suzuki et al., 2016), whose backbone sequence is flanked with minimal *attB* and *attP* sites and can be removed by phiC31 integrase-induced recombination either *in vivo* or *in vitro*. We first successfully removed the backbone of *sox10* Bi-FoRe KI alleles by injection of zebrafish codon optimized *phiC31* mRNA in zebrafish embryos (Fig. S8), which is consistent with previous reports of efficient *in vivo* site-specific excision of minimal *attB* and *attP* sites (Lister, 2010; Lu et al., 2011). For *in vitro* backbone removal, we prepared the *sox10* minicircle Bi-FoRe donor according to the minicircle production protocol (Lister, 2010; Lu et al., 2011) and used it for founder embryo injection. Our preliminary results showed that efficient and correct KI events were easily detected in minicircle donor injected embryos (Fig. 4A and 4B), and the ratio of mosaic F_0_ embryos increased by 14% compared with the full length Bi-FoRe donor (Table S2), which suggested that the smaller donor may be more efficient for NHEJ-mediated KI. In summary, our results showed that unfavorable bacterial backbone sequence could be efficiently eliminated either *in vivo* by *phiC31* mRNA injection or *in vitro* by minicircle DNA donor production. To our knowledge, this is the first report of the application of minicircle DNA technology in zebrafish.

In addition to integration into the target locus, donor vectors might also exhibit off-targeting insertions after injection into zebrafish embryos. Although we cannot exclude this possibility, we did not observe obvious ectopic expressions of fluorescent reporters in the F_0_ or F_1_ embryos in our experiments, indicating that random gene-trapping was undetectable. This may be due to the low random insertion efficiency of *in vivo* linearized donor vectors as previously reported (Auer et al., 2014), and in addition, the requirement for donors to be inserted into appropriate gene regions to ensure its expression. However, “silent” random insertions are still possible, which should be investigated in the future.

Besides what we have presented in this study, our FoRe and Bi-FoRe donor strategy can be further modified for expanded or improved applications. For example, a disease-related mutation could be substituted in the downstream CDS to generate disease models with allele expression tagging, or coding sequences of various protein tags could be fused in-frame with the remaining CDS for biochemical studies. Furthermore, since the CRISPR/Cas system, including Cas9 and Cas12a, has been widely and efficiently applied to many organisms (Mali et al., 2013; Zetsche et al., 2015; Hur et al., 2016; Moreno-Mateos et al., 2017), our bidirectional multi-function KI strategy should be applicable *in vivo* in other model systems, as well as *in vitro* in cell culture studies.

## Materials and methods

### Zebrafish husbandry

All the zebrafish used in this study were maintained at 28.5 °C in the zebrafish facility of Peking University with a 14 h/10 h light/dark cycle. The wild-type strain used was Tübingen (TU).

### Donor plasmid construction

To construct the FoRe donor for the *sox10* locus targeting the third intron, the *hEMX1* target site and the *lox66* site were linked by a 111-bp DNA fragment cloned from the pMD18-T vector (TAKARA, 6011) to avoid the disruption of the *lox66* sequence due to NHEJ-mediated DSB repair. This *hEMX1-linker-lox66* sequence was cloned into the pMD19-T simple vector (TAKARA, 3271). Then, the intron 3 and exon 4 sequence (without stop codon and 3′ UTR) downstream of the *sox10* target site (including the splicing acceptor) from the zebrafish genome was amplified and cloned into the above vector downstream to the *lox66* site. Then* T2A-tdTomato-SV40 pA* sequence was fused downstream to the *sox10* exon 4 (referred to as “Remaining CDS” in Fig. 1A). These elements aside from the *hEMX1* site were referred as the Forward component of the vector. Finally, the *lox71* sequence, the intron 3 sequence downstream of the *sox10* site and the first 98 bp of the *sox10* exon 4 sequence were ligated with *P2A-EGFP-BGH pA* and cloned into the above vector in reverse orientation downstream of the *SV40 pA* sequence, as the Reverse component of the vector. For the convenience of cloning/replacing other target gene sequence, KpnI and AvrII sites were introduced upstream and downstream, respectively, to the *sox10* sequence in the Forward component in forward direction, while SalI and EcoRV recognition sites were introduced upstream and downstream, respectively, to the *sox10* sequence in the Reverse component in reverse direction.

The above *sox10* FoRe donor was used as the basic vector to construct the FoRe donor for the* isl1* locus and the Bi-FoRe donor for the *sox10* locus. To construct the *isl1* FoRe donor, the *sox10 *FoRe donor was digested with KpnI and AvrII and used as the donor backbone. Then, the intron 3 sequence downstream of the *isl1* target site and the subsequent remaining downstream CDS without stop codon (referred to as “Remaining CDS” in Fig. S4B) were cloned into the backbone. The plasmid was then digested by SalI and EcoNI and ligated with the intron 3 sequence downstream of the* isl1* site and the first 17 bp of* isl1* exon 4 to generate the complete* isl1 *FoRe donor.

To construct the *sox10* Bi-FoRe donor, the minicircle plasmid pTUBB3-MC (gift from Juan Belmonte, Addgene plasmid #87112; http://n2t.net/addgene:87112; RRID: Addgene_87112) was used as the backbone after digestion with XmaI and BsrGI (Suzuki et al., 2016). The region spanning the *hEMX1* site and the entire Forward component was amplified from the *sox10* FoRe donor and ligated into the digested pTUBB3-MC plasmid, together with a *BGH pA* sequence. Then, the Reverse component from the *sox10* FoRe donor, including the sequence spanning the *BGH pA* through the *lox71* site, together with its upstream flanking *SV40 pA* sequence and downstream 70-bp sequence in the *sox10* FoRe donor (for protection from NHEJ-mediated sequence deletion), was amplified and cloned upstream of the *hEMX1* site to generate the full length *sox10* Bi-FoRe donor. For the production of the *sox10* Bi-FoRe minicircle donor vector, the above full length *sox10* Bi-FoRe donor was transformed into the ZYCY10P3S2T strain (System Biosciences), then bacteria amplification and plasmid extraction was performed as reported by Kay et al. (2010), except for adjusting the incubation volume to a level suitable for miniprep.

For kits and enzymes used in molecular cloning, initial T-A cloning was performed using Solution I (TAKARA), and the cloning of the subsequent fragments was performed using Gibson assembly according to the manufacture’s protocol (NEB, M5510AA). Restriction enzymes were ordered from NEB, and high-fidelity versions were adopted if available.

### Preparation of gRNA and the mRNAs encoding Cas9, Cre and phiC31

The zebrafish codon-optimized *Cas9* expression vector pT3TS-nCas9n was linearized by XbaI and used as the template for generating *Cas9* mRNA through *in vitro* transcription using the mMessage mMachine T3 kit (Ambion) (Jao et al., 2013).

The gRNAs were designed by using the CasOT program (http://casot.cbi.pku.edu.cn/) (Xiao et al., 2014). Forward oligonucleotides containing a T7 promoter, gRNA target site and partial gRNA scaffold sequences were designed for gRNA template synthesis through PCR amplification by using the pUC19-scaffold as the template (Chang et al., 2013), together with the universal reverse primer (5′-AAAAAAAGCACCGACTCGGTGCCAC-3′). Then, the gRNAs were synthesized by *in vitro* transcription with T7 RiboMAX™ Express Large Scale RNA Production System (Promega). The gRNA target sequences are listed in Table S1.

The *Cre* expression vector pX-T7-Cre (Li et al., 2019) was linearized by XbaI and used as the template for synthesizing *Cre* mRNA through *in vitro* transcription using the mMessage mMachine T7 kit (Ambion).

Zebrafish codon optimized *phiC31* (*zphiC31*) coding sequence was chemically synthesized (Ruibiotech Biotechnology, Beijing) and cloned to pT3TS plasmid to generate the pT3TS-zphiC31 plasmid. The plasmid was linearized by XbaI and used as template for *in vitro* transcription of *zphiC31* mRNA with the mMessage mMachine T3 kit (Ambion).

All the RNA products were purified by LiCl precipitation according to the manufacture’s protocol (Ambion).

### Microinjection of zebrafish embryos

To evaluate the indel efficiency of each target site, 1 nL of a solution containing 600 ng/μL *Cas9* mRNA and 100 ng/μL *gRNA* was injected into each one-cell stage zebrafish embryo. For knockin experiments, 1 nL of a solution containing 600 ng/μL *Cas9* mRNA, 100 ng/μL for each *gRNA* and 15 ng/μL of full length donor plasmid or 8 ng/μL of the minicircle donor (to ensure approximately equal amount of molecules for different donors) was injected into the animal pole of one-cell stage zebrafish embryos.

For Cre recombinase-induced inversion experiments, 10 pg *Cre* mRNA was injected into the vegetal pole of one-cell stage embryos to obtain mosaics, and 50 pg *Cre* mRNA was injected into the animal pole of one-cell stage embryos to achieve efficient Cre-mediated recombination.

For the experiments involving phiC31 function, 50 pg *zphiC31* mRNA was injected into the animal pole of one-cell stage embryos to induce recombination of minimal* attB* and *attP in vivo*.

### Restriction endonuclease assay to evaluate indel efficiencies of the Cas9 target sites

To prepare genomic DNA, five 24 hpf embryos were collected in each tube and lysed by 20 μL 50 mmol/L NaOH solution at 95 °C for 15 min, then neutralized by 2 μL 1 mol/L Tris-HCl (pH = 8.0). Then, 1 μL of the genomic DNA extract was used as the template to PCR amplify the region spanning the target sites using relevant primers (Table S7). The PCR products were then digested by corresponding restriction endonucleases and analyzed by agarose gel electrophoresis. The indel efficiency was estimated by the ratio of undigested products.

### Junction PCR and Sanger sequencing

Genomic DNA of 48–72 hpf F_1_ embryos was extracted by NaOH lysis as described above. Then, genome pool of corresponding embryos (3–5 individuals) was used as template to PCR amplify the 5′ and 3′ junction fragments of the KI alleles using the appropriate primers (Table S7). The PCR products were directly sent for Sanger sequencing.

### RNA extraction, cDNA synthesis and RT-PCR analysis

RNA was isolated from 10 embryos at 2–3 dpf (days post-fertilization) using Trizol reagent (Invitrogen) according to the manufacture’s protocol. 1 μg of total RNA was used for cDNA synthesis by reverse transcription using 5X All-In-One RT MasterMix (Applied Biological Materials). 1 μL of the resulting cDNA was used as template for PCR amplification with corresponding primers (Table S7), and *gapdh* was used as internal reference. The predicted length of the PCR products generated by sRT or iRT primer pairs is 467 bp and 509 bp, respectively.

### Imaging and processing

For general imaging, zebrafish embryos were anesthetized with 0.02% tricaine (ethyl 3-aminobenzoate methanesulfonate salt, Sigma), positioned in 3% methylcellulose (Sigma), and imaged under a compound microscope (AXIO Imager Z1; Zeiss) equipped with AxioCam MRm (Zeiss). The images were processed by the AxioVision Rel.4.8 software. Confocal imaging was performed using a LSM 710 confocal microscope (Zeiss) with a 10× or 20× water immersion objective, and Z-stack images were acquired with a 5 to 7 μm resolution and processed by the ZEN 2009 imaging software. Lightsheet imaging was performed using a Lightsheet Z1 microscope (Zeiss) with a 10X objective, and the images were processed by the ZEN 2014 SP1 imaging software. The embryos used for imaging in all the *isl1* KI experiments as well as *sox10* KI experiments in Fig. S2B and S2C were pretreated with 0.0045% PTU (1-Phenyl-2-thiourea, Sigma) dissolved in fish water for inhibition of the pigmentation.

## Electronic supplementary material

Below is the link to the electronic supplementary material.

Supplementary material 1 (PDF 12440 kb)

Supplementary movie 1 (MP4 8853 kb)
