# Supplementary material for: Bi-FoRe: an efficient bidirectional knockin strategy to generate pairwise conditional alleles with fluorescent indicators
Source: Protein Cell. 2020 Jul 17;12(1):39–56. doi: 10.1007/s13238-020-00747-1 (PMC7815861; doi:10.1007/s13238-020-00747-1)
Supplement: Supplementary file 1 — Supplementary material 1 (PDF 12440 kb) [file 13238_2020_747_MOESM1_ESM.pdf]

Supplementary figure 1

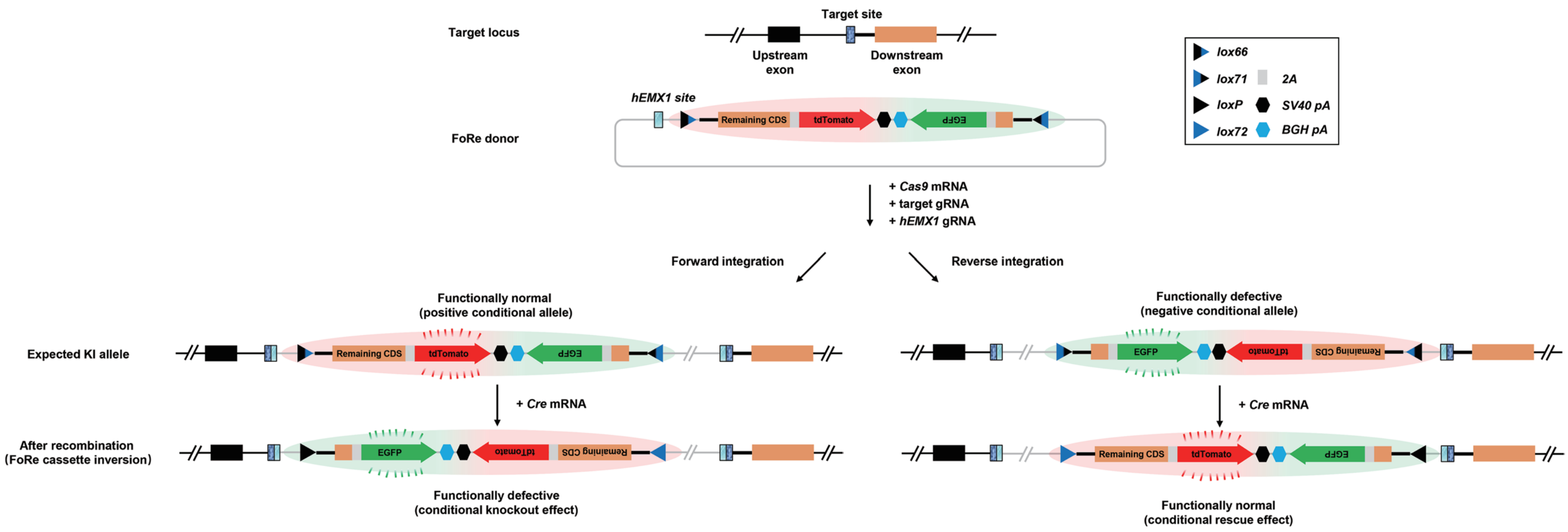

Supplementary figure 2

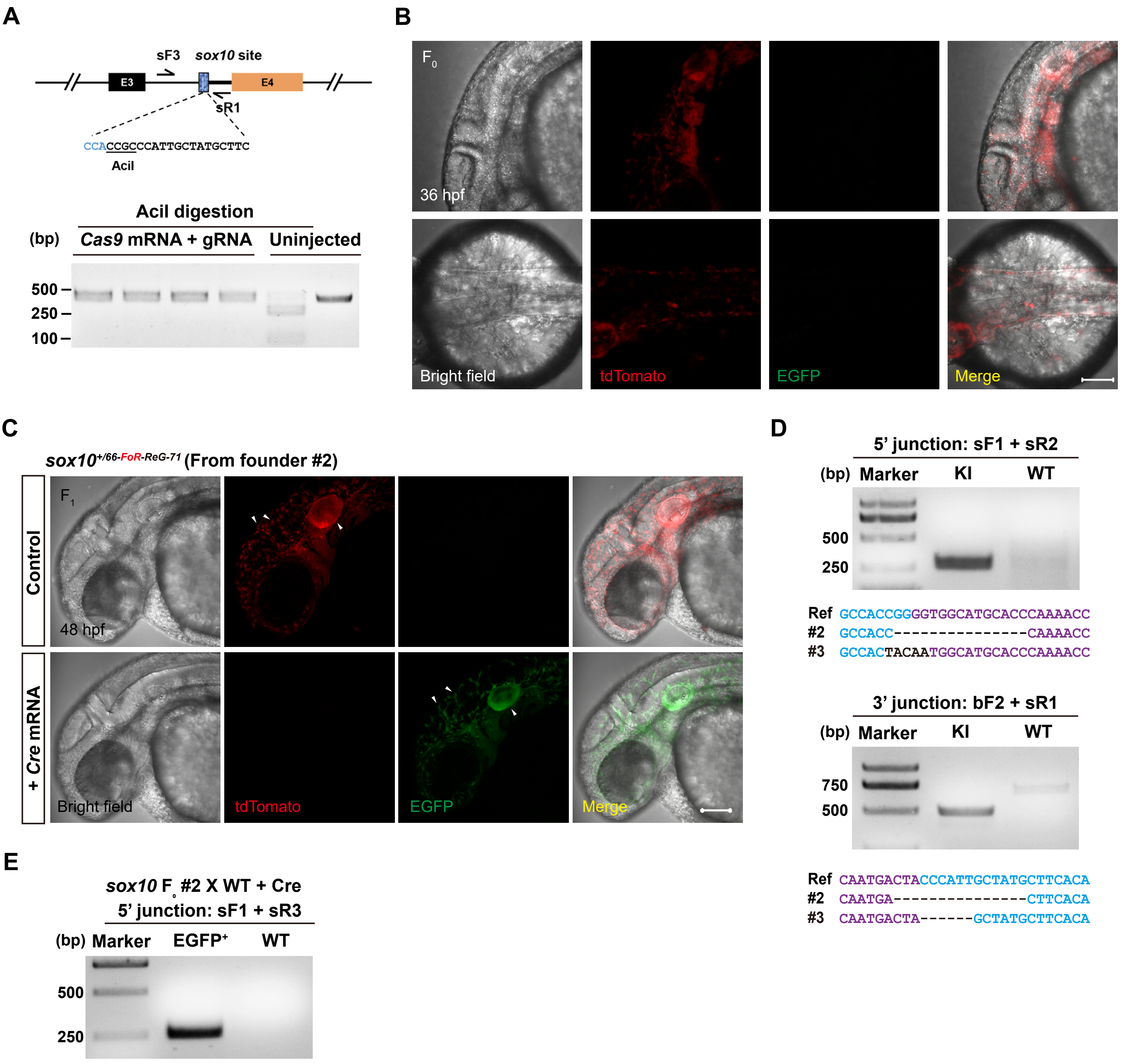

Supplementary figure 3

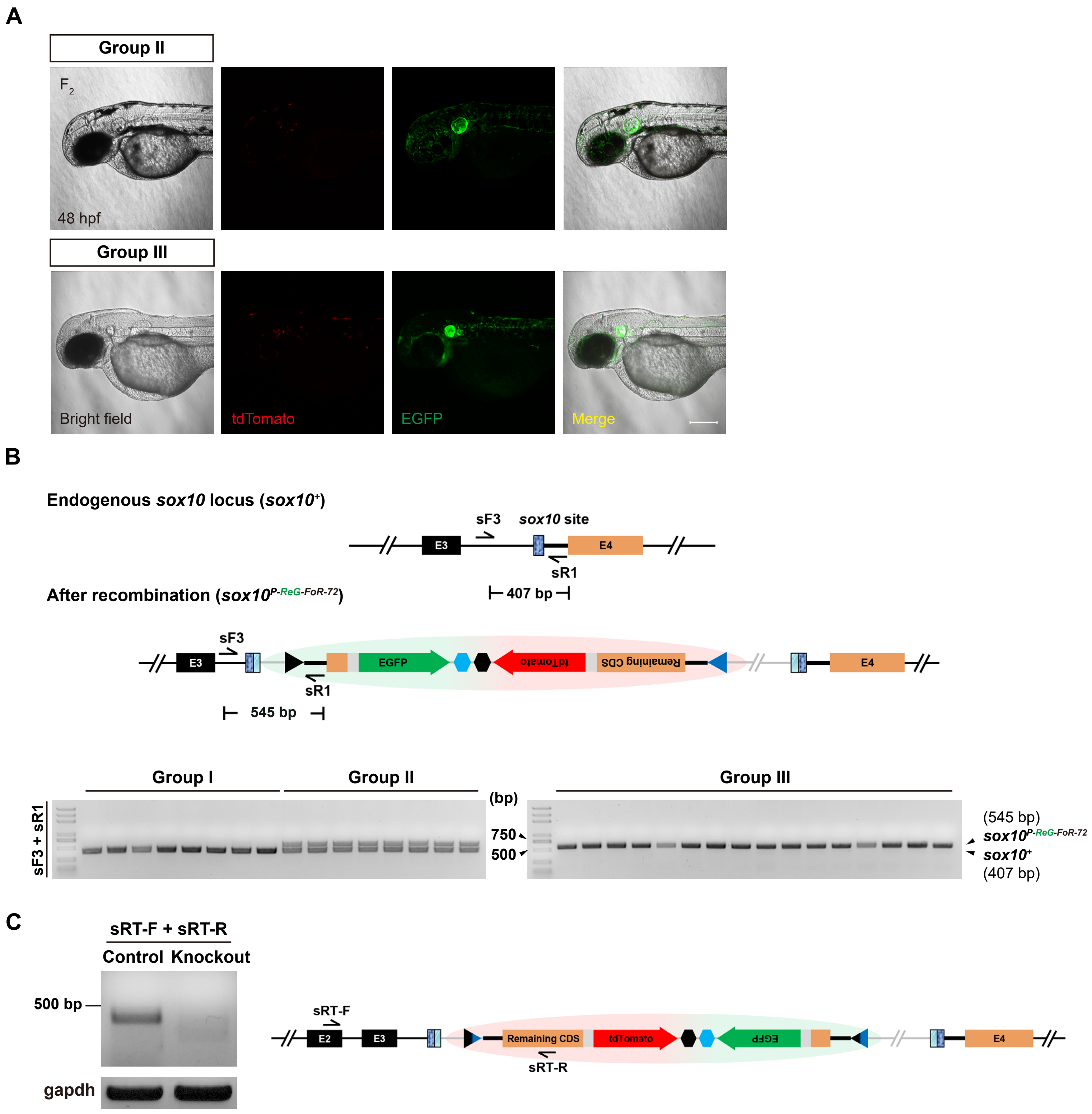

Supplementary figure 4

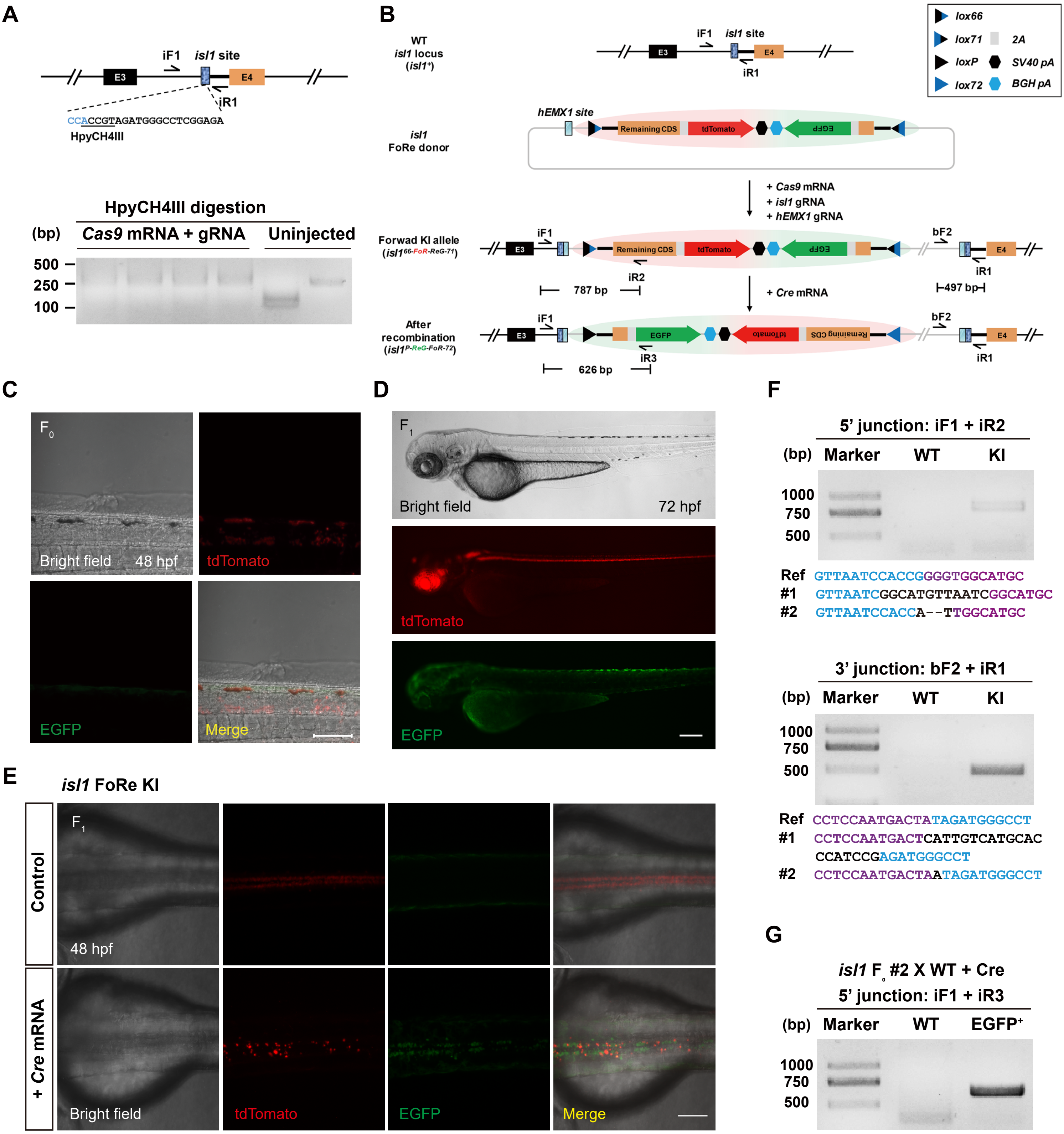

Supplementary figure 5

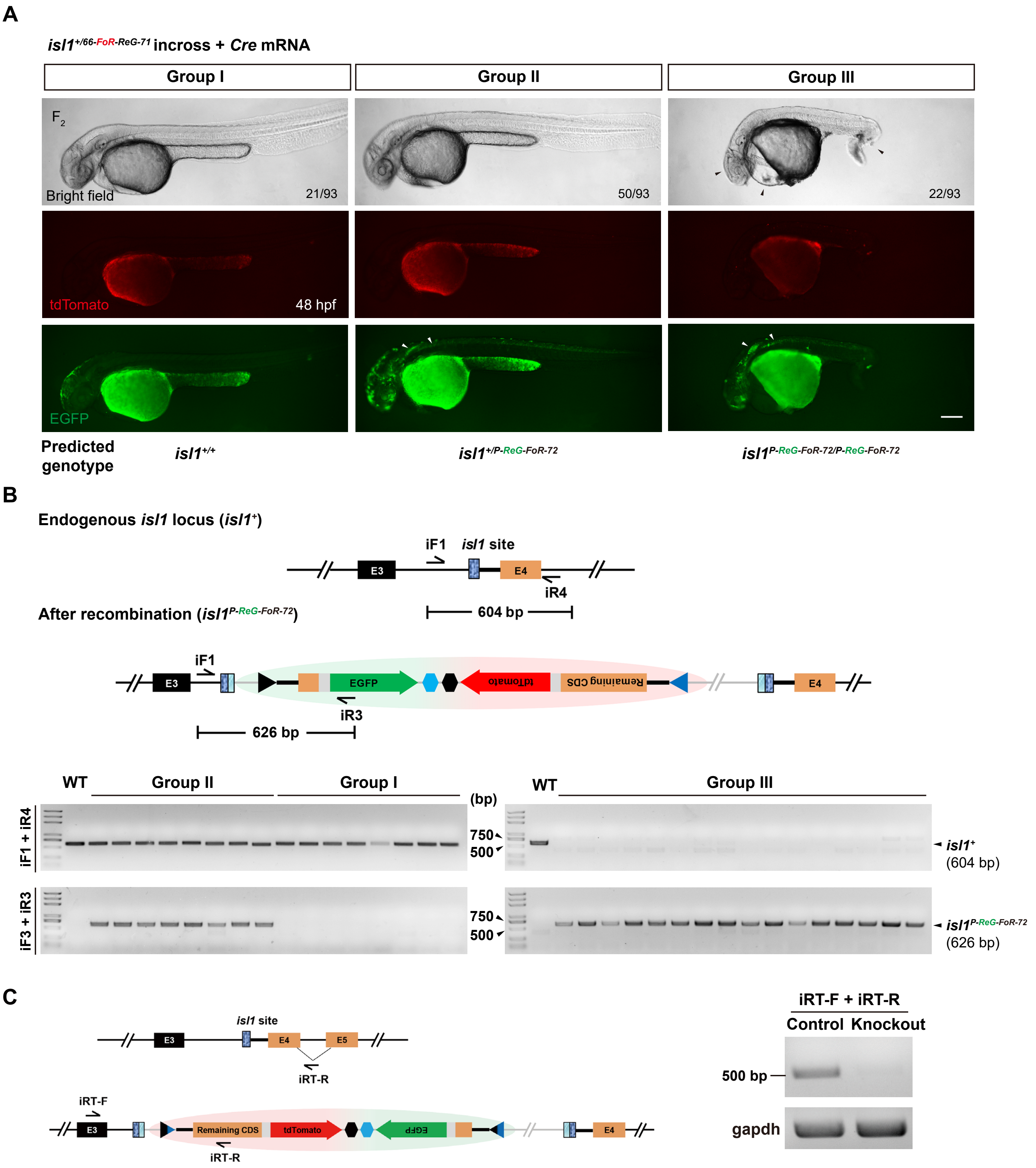

Supplementary figure 6

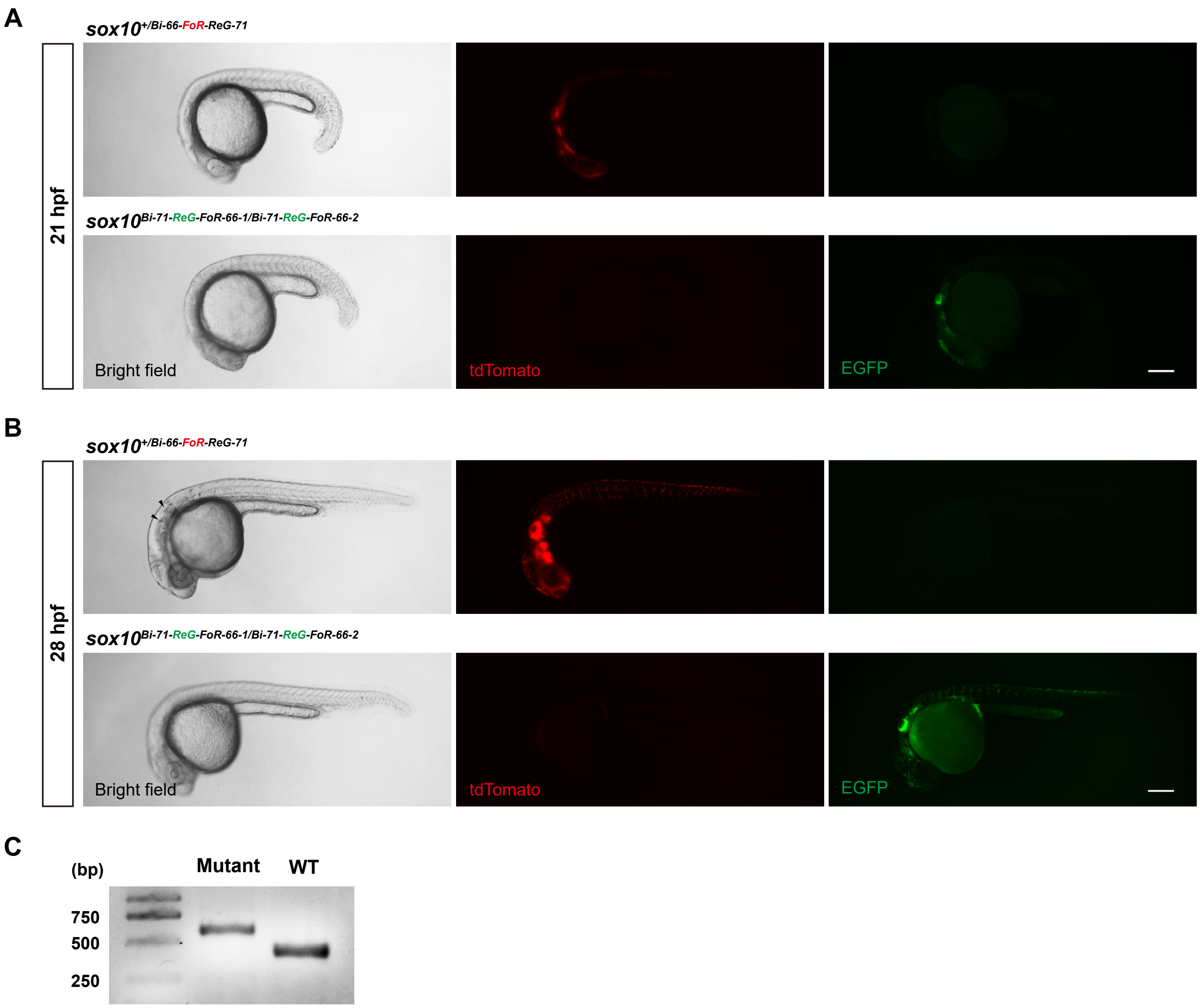

Supplementary figure 7

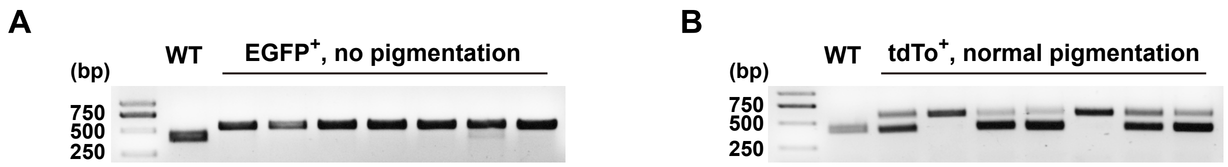

Supplementary figure 8

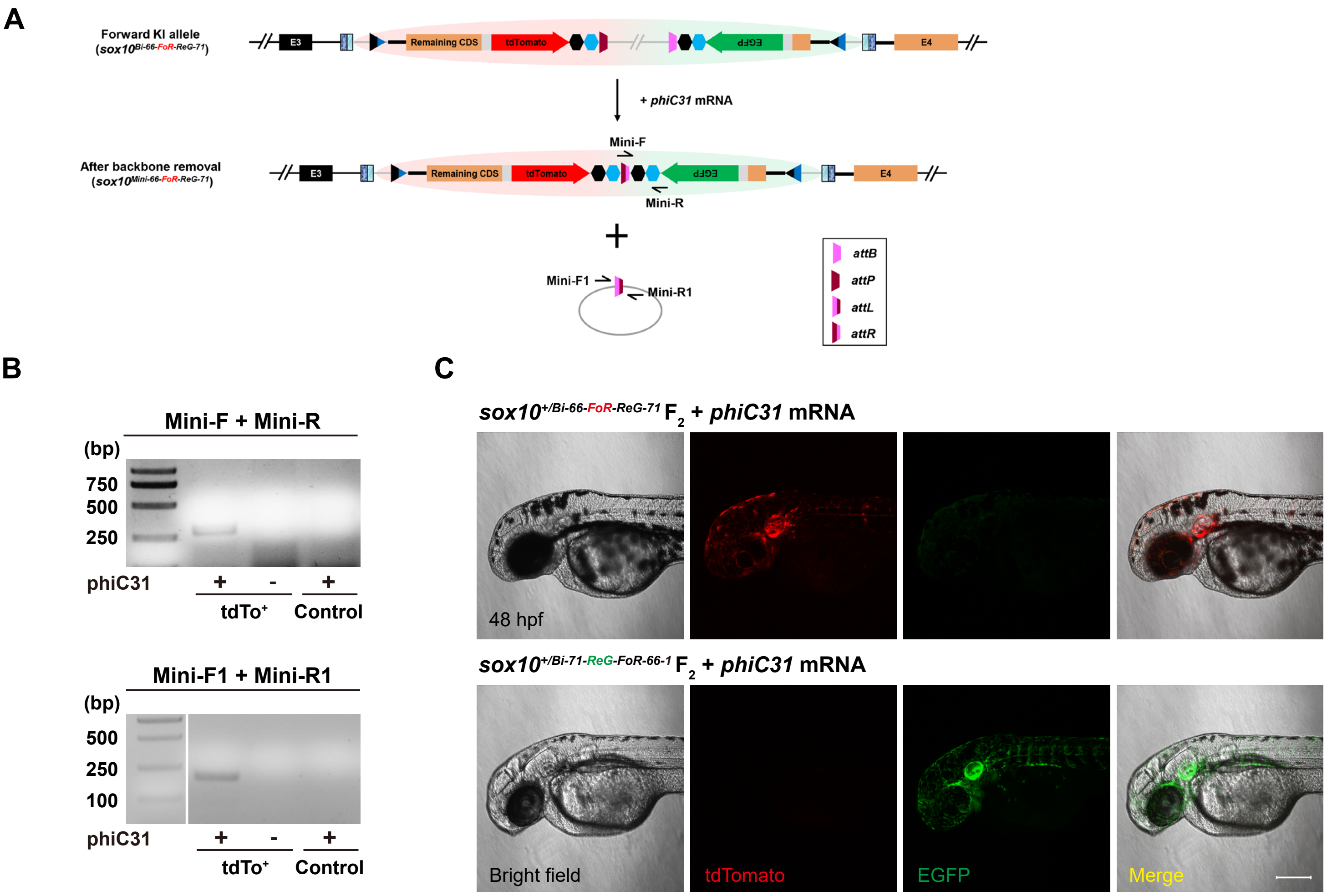

Supplementary figure 9

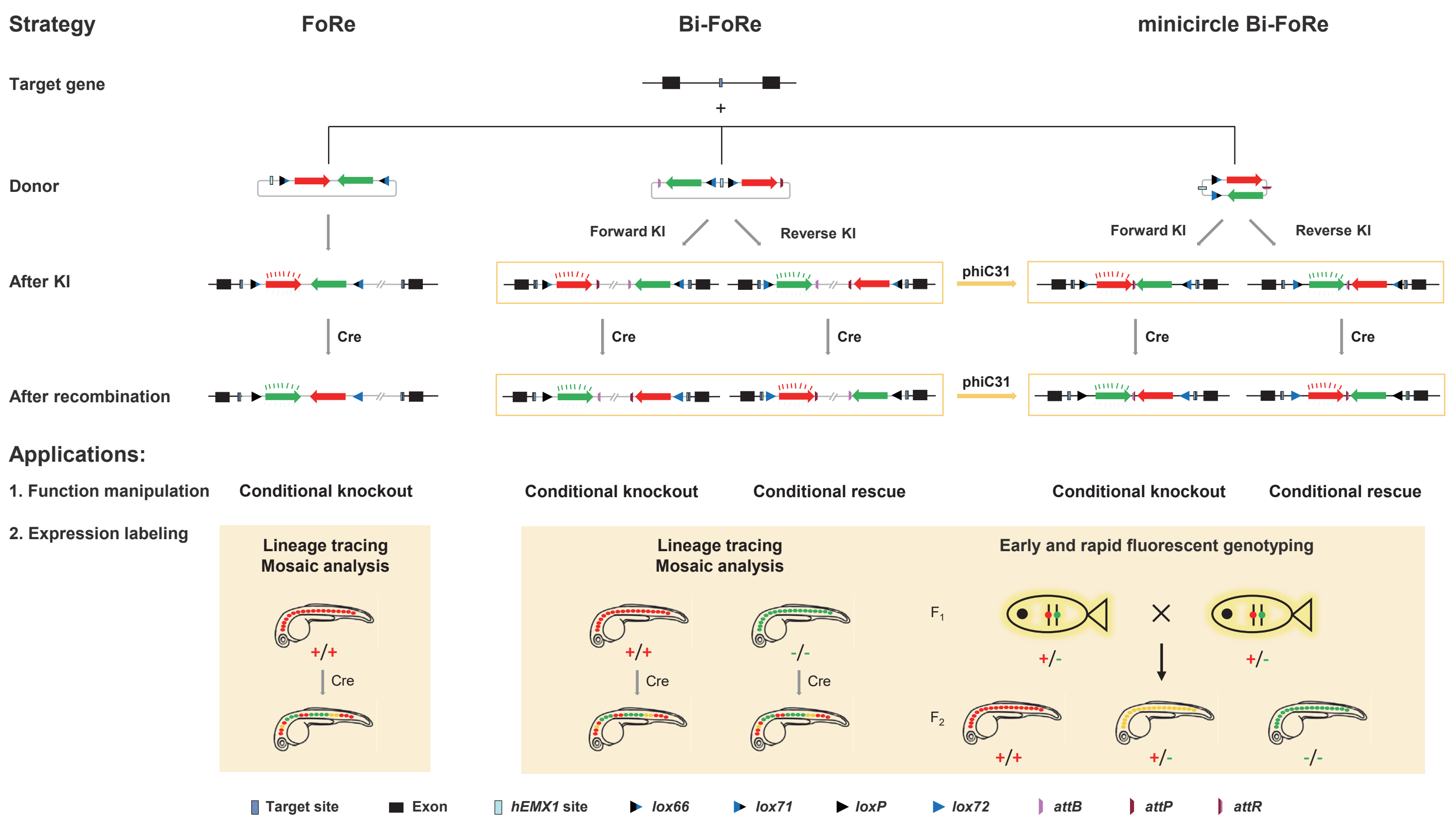

Supplementary Figure Legends

Supplementary figure 1. Schematic diagram of the rationale and design of the bidirectional KI strategy based on the dual-function FoRe donor consisting of two functional parts. The Forward part for maintaining target gene function is highlighted by red shadow, while the Reverse part for disrupting target gene function is highlighted by green shadow. *tdTomato* and *EGFP* fluorescent reporters were fused in-frame with the coding sequence of the target gene in the Forward part and the Reverse part, respectively, to label the expression of the corresponding KI allele. The two parts were arranged in a back-to-back opposite orientation and flanked by a *lox66* site and a *lox71* site in an opposite orientation, to ensure conditional manipulation of the KI alleles by Cre recombinase. The expected results of targeted integration of the FoRe donor in both directions as well as effects of Cre-induced FoRe cassette inversion are shown.

Supplementary figure 2. Generation and evaluation of the zebrafish *sox10* FoRe KI alleles. (A) The position and sequence of the *sox10* intron CRISPR/Cas system target site designed for the KI experiments. The PAM is shown in blue. Indel efficiency was estimated by PCR and AciI restriction endonuclease digestion. (B) Z-stack confocal images of two representative *sox10* FoRe donor KI founder embryos at 36 hpf. Scale bar, 100 μm. (C) Z-stack confocal images of 48 hpf F_1 larvae from an outcross of the *sox10* FoRe donor KI founder #2 with (+ *Cre* mRNA) or without (Control) *Cre* mRNA injection, showing fluorescence in the otic vesicle and pigment cells (white arrowheads). Scale bar, 100 μm. (D) Junction PCR and direct sequencing results of *sox10* FoRe donor KI F_1 embryos from outcross of F_0 #2 or #3. Blue and purple text represent the predicted endogenous genomic sequence and exogenous donor sequence, respectively, while black characters indicate sequence alterations at the junction site aligned to the reference sequence (Ref). The color code for sequence text remains the same for all the other figures concerning sequence alignments. KI: pooled genomic DNA template of *sox10* FoRe KI embryos from outcross of F_0 #2. WT: pooled genomic DNA template of wild-type embryos. (E) 5' junction PCR result of the F_1 embryos from

outcross of F_0 #2 bearing the *sox10*$^{+/66\text{-}FoR\text{-}ReG\text{-}71}$ allele with WT fish and showing green fluorescent signals after *Cre* mRNA injection. EGFP$^+$: pooled genomic DNA template from the EGFP-positive F_1 progeny after *Cre* mRNA injection. WT: pooled genomic DNA template of wild-type embryos.

Supplementary figure 3. Cre recombinase dependent *sox10* inactivation via *sox10* FoRe KI alleles. (A) Higher magnification images of embryos from incross of *sox10*$^{+/66\text{-}FoR\text{-}ReG\text{-}71}$ heterozygotes (Fig. 1C, Groups II and III). Scale bar, 200 μm. (B) Genotyping results of the individual *Cre* mRNA-injected embryos from panel A. A 407-bp fragment derived from the endogenous wild-type allele or a 545-bp fragment derived from the recombinant KI allele could be PCR amplified using primers sF3 and sR1. (C) RT-PCR results using cDNA of embryos from an incross of the *sox10*$^{+/66\text{-}FoR\text{-}ReG\text{-}71}$ heterozygotes with or without *Cre* mRNA injection. Control: embryos showing red fluorescence without *Cre* mRNA injection; Knockout: embryos showing green fluorescence and no pigmentation after *Cre* mRNA injection, similar to Group III from Fig. 1C.

Supplementary figure 4. Generation and evaluation of the zebrafish *isl1* FoRe KI alleles. (A) The position and sequence of the *isl1* intron CRISPR/Cas system target site designed for the KI experiments. The PAM is shown in blue. Indel efficiency was estimated by PCR and HpyCH4III restriction endonuclease digestion. (B) Schematic diagram of the KI strategy at the *isl1* locus based on the dual-function FoRe donor consisting of two parts. The Forward part is highlighted by red shadow and used for maintaining the function of the *isl1* gene, and the Reverse part is highlighted by red shadow and used for disruption of the *isl1*. The *isl1* target site is shown in dark blue, and the *hEMX1* site is shown in light blue, located upstream to the Forward part in the donor. (C) Z-stack confocal images of a representative *isl1* FoRe donor KI founder embryo at 48 hpf. Scale bar, 200 μm. (D) Representative red fluorescent expression pattern of an *isl1* FoRe donor KI F_1 embryo from outcross of F_0 #2 in eyes and motor neurons at 72 hpf. Scale bar, 200 μm. (E) Z-stack confocal images of 48 hpf F_1 larvae

from an outcross of an *isl1* FoRe KI founder (#2) with (+ *Cre* mRNA) or without (Control) 10 pg *Cre* mRNA injection, showing fluorescent signals in the trunk motor neurons. The embryos are shown in dorsal view with anterior to the left. Scale bar, 100 µm. (F) Junction PCR and direct sequencing results of *isl1* FoRe donor KI F_1 embryos from outcross of F_0 #1 or #2. KI: pooled genomic DNA template of the F_1 embryos from outcross of F_0 #2. WT: pooled genomic DNA template of wild-type embryos. (G) 5' junction PCR result of F_1 embryos from outcross of F_0 #2 bearing the *isl1*$^{+/66\text{-}FoR\text{-}ReG\text{-}71}$ allele with WT after *Cre* mRNA injection. EGFP$^+$: pooled genomic DNA template from the F_1 progeny that were EGFP-positive after *Cre* mRNA injection. WT: pooled genomic DNA template of wild-type embryos.

Supplementary figure 5. Cre recombinase dependent *isl1* inactivation via *isl1* FoRe KI alleles. (A) Phenotype analysis of the 48 hpf F_2 embryos from the incross of *isl1*$^{+/66\text{-}PoR\text{-}ReG\text{-}71}$ heterozygotes (derived from F_0 #2) after the injection of 50 pg *Cre* mRNA at the one-cell stage. All injected embryos showed little tdTomato fluorescent signal. Among these embryos, 22.6% (21/93) showed normal development and no fluorescence (Group I: EGFP$^-$, no phenotype), these embryos were likely *isl1*$^{+/+}$. 53.8% (50/93) of the injected embryos also developed normally, but were positive for *EGFP* fluorescent signal (Group II: EGFP$^+$, no phenotype), whose genotype is expected to be *isl1*$^{+/P\text{-}ReG\text{-}FoR\text{-}72}$ heterozygous. 23.6% (22/93) exhibited several distinct phenotypes, including smaller head and reduced body size, abnormal heart, and curved body axis (black arrowheads). All of these embryos were positive for EGFP expression (Group III: EGFP$^+$, obvious phenotype), suggesting that these embryos are likely *isl1*$^{P\text{-}ReG\text{-}FoR\text{-}72/P\text{-}ReG\text{-}FoR\text{-}72}$ homozygous. White arrowheads indicate EGFP expression in trunk neurons. Scale bar, 200 µm. (B) Genotyping results of the individual *Cre* mRNA-injected embryos from A. A 604-bp endogenous wild-type fragment could be amplified by using the iF1 and iR4 primer pair, and a 626-bp recombinant KI fragment could be amplified by using the iF3 and iR3 primer pair. (C) RT-PCR results using the cDNA of embryos from an incross of the *isl1*$^{+/66\text{-}FoR\text{-}ReG\text{-}71}$ heterozygotes with or without *Cre* mRNA injection. Control: embryos showing red fluorescence without *Cre* mRNA

injection; Knockout: embryos showing phenotype and green fluorescence after *Cre* mRNA injection, similar to Group III from Fig. S5A.

Supplementary figure 6. Fluorescent reporter expression and phenotype analyses of embryos bearing *sox10* Bi-FoRe KI alleles at early developmental stages. (A) Bright-field and fluorescence micrographs of a *sox10* $^{+/Bi\text{-}66\text{-}FoR\text{-}ReG\text{-}71}$ F_2 embryo (upper panel) from the outcross of *sox10*$^{+/Bi\text{-}66\text{-}FoR\text{-}ReG\text{-}71}$ with wild type, and a *sox10*$^{Bi\text{-}71\text{-}ReG\text{-}FoR\text{-}66\text{-}1/Bi\text{-}71\text{-}ReG\text{-}FoR\text{-}66\text{-}2}$ embryo (lower panel) from the outcross between #1 and #7 founders at 21 hpf, captured using a Zeiss AXIO Imager Z1 microscope. (B) The embryos from panel A imaged at 28 hpf. Black arrowheads indicate melanocytes. (C) Genotyping results of the embryo used for imaging in A and B which showed no melanocytes by 28 hpf. A 407-bp fragment from the endogenous wild-type allele or a ~560-bp fragment from the KI allele could be amplified using primers sF3 and sR1. Mutant: genome of the embryo with no melanocytes by 28 hpf. WT: pooled genome of wild-type embryos.

Supplementary figure 7. Genotyping evaluation on the conditional effects of the *sox10* Bi-FoRe KI alleles. (A) Genotyping results of seven individual *Cre* mRNA-injected embryos lacking pigments and showing EGFP expression (EGFP$^+$), similar to the one shown in Figure 3B. (B) Genotyping results of seven individual *Cre* mRNA-injected embryos with normal pigmentation and tdTomato expression (tdTo$^+$), similar to the one shown in Figure 3F. A 407-bp fragment corresponding to the endogenous wild-type allele or a ~560-bp fragment corresponding to the KI allele could be amplified using primers sF3 and sR1.

Supplementary figure 8. Elimination of the plasmid backbone by phiC31-mediated *in vivo* recombination. (A) Schematic diagram of the *in vivo* elimination of the plasmid backbone in the *sox10* Bi-FoRe KI alleles by phiC31-mediated recombination. Note that the sequence of the Mini-F primer is within attR and is attR-specific. (B) Junction PCR detection of phiC31-mediated recombination products in the F_2 embryos obtained from outcross of *sox10*$^{+/Bi\text{-}66\text{-}FoR\text{-}ReG\text{-}71}$ F_1 with or without *phiC31*

mRNA injection. tdTo^{+}: pooled genome from the embryos with tdTomato expression; Control: pooled genome from the embryos with no tdTomato expression. The predicted length of PCR products amplified using Mini-F/Mini-R or Mini-F1/Mini-R1 primer pairs are 278 bp and 169 bp, respectively. (C) Z-stack confocal images of the *sox10*$^{+/Bi\text{-}66\text{-}FoR\text{-}ReG\text{-}71}$ or *sox10*$^{+/Bi\text{-}71\text{-}ReG\text{-}FoR\text{-}66\text{-}1}$ embryos injected with *phiC31* mRNA. After injection, both the red and green fluorescence expression patterns remain unchanged. Scale bar, 200 μm.

Supplementary figure 9. Comparison between our FoRe and Bi-FoRe KI strategies.

Supplementary Movie Legend

Supplementary movie 1. Reconstructed movie from time-lapse lightsheet imaging of a *sox10* mosaic embryo from incross of *sox10*$^{66\text{-}FoR\text{-}ReG\text{-}71/66\text{-}FoR\text{-}ReG\text{-}71}$ homozygotes after vegetal pole injection of 10 pg *Cre* mRNA, recorded from 24 hpf to 36.5 hpf.

Supplementary table 1. The gRNA target sequences used in this study

Target site	Target sequence*
sox10	CCACCGCCCATTGCTATGCTTC (reverse strand)
isl1	CCACCGTAGATGGGCCTCGGAGA (reverse strand)
hEMX1	GTCACCTCCAATGACTAGGGTGG (forward strand) (Lin et al., 2014)

*The underlined sequence represents PAM.

Reference: Lin, S., Staahl, B.T., Alla, R.K., and Doudna, J.A. (2014). Enhanced homology-directed human genome engineering by controlled timing of CRISPR/Cas9 delivery. Elife *3*, e04766.

Supplementary table 2. Knockin efficiencies in F_0 embryos

Target site	Donor	Ratio of fluorescence-positive F_0 embryos
sox10	FoRe	38/93 (40.9%)
isl1	FoRe	13/79 (16.5%)
sox10	Bi-FoRe	69/135 (51.1%) (forward & reverse)
sox10	minicircle Bi-FoRe	95/147 (64.6%) (forward & reverse)

Supplementary table 3. Germline mosaicism of the *sox10* FoRe donor KI in each positive F_0

F_0 (founder)	Sex	Number of *tdTomato*-positive F_1 embryos	Number of total F_1 progeny	F_0 mosaicism (ratio of *tdTomato*-positive F_1 embryos)	Allele name
**#2**	Male	9	16	56.3%	$sox10^{66\text{-}FoR\text{-}ReG\text{-}71}$
#3	Male	13	26	50.0%	
#4	Female	2	198	1.0%	
#6	Male	15	74	20.3%	

Supplementary table 4. Germline mosaicism of the *isl1* FoRe donor KI in each positive F_0

F_0 (founder)	Sex	Number of *tdTomato*-positive F_1 embryos	Number of total F_1 progeny	F_0 mosaicism (ratio of *tdTomato*-positive F_1 embryos)	Allele name
#1	Male	3	42	7.14%	
**#2**	Male	40	122	32.79%	$isl1^{66\text{-}FoR\text{-}ReG\text{-}71}$
#3	Male	3	31	9.68%	

Supplementary table 5. Germline mosaicism of the *sox10* Bi-FoRe donor forward KI in each positive F_0

F_0 (founder)	Sex	Number of *tdTomato*-positive F_1 embryos	Number of total F_1 progeny	F_0 mosaicism (ratio of *tdTomato*-positive F_1 embryos)	Allele name
**#3**	Male	9	44	20.5%	$sox10^{Bi\text{-}66\text{-}FoR\text{-}ReG\text{-}71}$
#4	Male	3	174	1.7%	
#5	Male	14	47	27.7%	
#6	Male	15	89	16.9%	

Supplementary table 6. Germline mosaicism of the *sox10* Bi-FoRe donor reverse KI in each positive F_0

F_0 (founder)	Sex	Number of *EGFP*-positive F_1 embryos	Number of total F_1 progeny	F_0 mosaicism (ratio of *EGFP*-positive F_1 embryos)	Allele name
**#1**	Male	18	86	20.9%	$sox10^{Bi\text{-}71\text{-}ReG\text{-}FoR\text{-}66\text{-}1}$
#2	Male	18	232	7.8%	
#5	Male	13	47	27.7%	
#6	Male	30	89	33.7%	
**#7**	Female	17	60	28.3%	$sox10^{Bi\text{-}71\text{-}ReG\text{-}FoR\text{-}66\text{-}2}$
#8	Female	34	121	28.1%	

Supplementary table 7. The primers used for PCR and RT-PCR analyses

Primer name	Primer sequence
sF1	CGGTATATAACGGTAGGCTAC
sR1	GAAACTATACACTAGGTCCA
bF2	CCTGCGTTATCCCCTGATTC
sR2	GGTACCTTTCCATTACAAGCGCCGCTC
sF3	TTGTTCTGTAGACTATCTAACAA
sR3	GAAGCATAGCGTCGACATAAC
sF4	ATGTATGCTATACGAACGGTA
sR4	TGTTGAACAATTTGCCATCGT
sRT-F	CGGATCAATATCCGCACCTG
sRT-R	AGACGATGAGGCGGAGCTTC
iF1	CCGGTCTCTTAAACAGATC
iR1	GCAAAGAGCTGTTGTATGATG
iR2	GTCATCCCCTGGATGTTCGTTT
iR3	CGCTGAACTTGTGGCCGTTTAC
iR4	CTGAAGCGCCACCAGTCTTAC
iRT-F	AGATGTGTGGCGTGTAGTCG
iRT-R	CCGTCGTGTCTCTCTGGACT
Mini-F	AACTGGGGTAACCTTTGGGCT
Mini-R	GCTGGCAACTAGAAGGCACAG
Mini-F1	ACTCGAGATCCACTAGAGTG
Mini-R1	CCCAGATAAACTCAATGATGATG
gapdhRT-F	GGTATTAACGGATTCGGTCGC
gapdhRT-R	GGCCATACCAGTAAGCTTGCC
